# Lipid Encapsulation Provides Insufficient Total-Tract Digestibility to Achieve an Optimal Transfer Efficiency of Fatty Acids to Milk Fat

**DOI:** 10.1371/journal.pone.0164700

**Published:** 2016-10-14

**Authors:** Melissa Bainbridge, Jana Kraft

**Affiliations:** University of Vermont, Department of Animal and Veterinary Sciences, 570 Main Street, Burlington, VT 05405, United States of America; Laval University, CANADA

## Abstract

Transfer efficiencies of rumen-protected n-3 fatty acids (FA) to milk are low, thus we hypothesized that rumen-protection technologies allow for biohydrogenation and excretion of n-3 FA. The objectives of this study were to i) investigate the ruminal protection and post-ruminal release of the FA derived from the lipid-encapsulated echium oil (EEO), and ii) assess the bioavailability and metabolism of the EEO-derived FA through measuring the FA content in plasma lipid fractions, feces, and milk. The EEO was tested for rumen stability using the *in situ* nylon bag technique, then the apparent total-tract digestibility was assessed *in vivo* using six Holstein dairy cattle. Diets consisted of a control (no EEO); 1.5% of dry matter (DM) as EEO and 1.5% DM as encapsulation matrix; and 3% DM as EEO. The EEO was rumen-stable and had no effect on animal production. EEO-derived FA were incorporated into all plasma lipid fractions, with the highest proportion of n-3 FA observed in cholesterol esters. Fecal excretion of EEO-derived FA ranged from 7–14%. Biohydrogenation products increased in milk, plasma, and feces with EEO supplementation. In conclusion, lipid-encapsulation provides inadequate digestibility to achieve an optimal transfer efficiency of n-3 FA to milk.

## Introduction

Over the past two decades, consumers have become increasingly conscious about the nutritional value of foods and their components with respect to health maintenance and disease prevention. Animal foods make a significant contribution to the daily diet in Western societies and dietary guidelines advise limiting the intake of animal fats, particularly ruminant-derived, since their higher content of saturated fatty acids (SFA) has been linked to various chronic diseases[1]. Although the purported link between SFA, derived from ruminant fats (*i*.*e*., meat and dairy), and the incidence of chronic diseases continues to be debated, there has been heightened research interest in modifying ruminant fats to meet consumer preferences and align with recommendations by public health authorities, nutritionists, and health-care providers. Ruminant dairy fats are comprised of approximately 65–70% SFA and only 2–4% polyunsaturated fatty acids (PUFA) [2] because of the extensive microbial biohydrogenation of feed-derived unsaturated fatty acids (UFA) in the rumen [3]. Thus, efforts to alter the fatty acid (FA) composition of dairy lipids include the reduction of SFA while increasing the content of UFA, especially PUFA and n-3 FA. Various vegetable and marine oils, but also oilseeds, have been used to supplement the dairy cows’ ration as a means to increase the concentration of UFA in milk fat. However, when unprotected oils are fed to cows, ruminal bacteria extensively hydrolyze and biohydrogenate the dietary lipids, resulting in marginal increases in passage to the small intestine [4,5]. Moreover, detrimental side effects, such as altered rumen biohydrogenation pathways associated with decreased fiber digestion, dry matter intake (DMI), and milk fat depression, have been observed [6,7]. Bypassing ruminal biohydrogenation and degradation of UFA may be achieved through the utilization of rumen-inert (*i*.*e*., calcium salts) or rumen-protected (*i*.*e*., fatty acid amides, formaldehyde-treated, lipid-encapsulated) oils [8] that may represent an opportunity to achieve a desired consistent milk fatty acid composition. Yet, these products have produced wide-ranging and inconsistent results [9]. We previously supplemented a total-mixed ration diet of mid-lactating Holstein cows with lipid-encapsulated echium oil (EEO) at 1.5 and 3.0% of dry matter (DM) to enhance the content of bioactive fatty acids (FA) in milk fat [10]. The protected supplement contained 25% of echium oil rich in α-linolenic acid (18:3 *c*9,*c*12,*c*15, ALA), steariodonic acid (18:4 *c*6,*c*9,*c*12,*c*15, SDA), and γ-linolenic acid (18:3 *c*6,*c*9,*c*12, GLA). Although the content of ALA, SDA, and GLA in milk fat increased, relatively low transfer efficiencies into milk fat were observed (ALA: 3.4–3.9%; SDA: 4.1–4.7%; and GLA: 2.8–3.0%). We hypothesize that either i) the EEO was not rumen stable and FA losses occurred as result of bacterial biohydrogenation, ii) EEO did not become available for absorption and utilization in the small intestine, or iii) EEO-derived FA were incorporated into plasma lipid fractions that are less available to the mammary gland. The objectives of this study were to i) investigate the ruminal protection and post-ruminal release of the FA derived from the EEO, and ii) assess the bioavailability and metabolism of the EEO-derived FA through measuring the FA content in plasma lipid fractions, feces, and milk.

## Materials and Methods

### Experiment 1

#### *In situ* Nylon Bag Procedure

All procedures involving animals were approved under the University of Vermont Institutional Animal Care and Use Committee (Protocol # 12–036). EEO was first evaluated for rumen stability using the *in situ* nylon bag technique. Echium oil (*Echium plantagineum*) was purchased from Technology Crops International (Winston-Salem, NC, USA) and micro-encapsulated with a hydrogenated vegetable oil by Jefo (Saint-Hyacinthe, QC, Canada) using a spray cooling method with a prilling atomizer. The final encapsulated product contained 25% echium oil.

EEO and a wheat straw (*i*.*e*., control) were incubated in the rumen of two rumen-cannulated non-lactating dairy cattle in a repeated measures design (x3). Five g of EEO and 2.5 g of wheat straw were weighed into individual nylon bags measuring 10 x 20 cm, with a pore size of 50 μm (ANKOM Technology; Macedon, NY). Three replicates of each sample were performed per time point per cow. Bags were heat sealed and distributed among three mesh retaining bags (Household Essentials, Hazelwood, MO) attached to a weight to control the location within the rumen. Samples were removed at time points 2, 4, 8, 16, 48, and 72 hours, and machine-rinsed, using the cold water cycle, until there was no color remaining in the rinse water. Zero-hour bags did not enter the rumen, but were subjected to the same procedures as the other bags (i.e., washing). The bags were dried at 65°C, placed in a desiccator until cool, and weighed to determine percent dry matter disappearance.

### Experiment 2

#### Animals and Experimental Design

Six lactating Holstein dairy cattle at 188 ± 43 days in milk (DIM) were used to assess the digestibility and incorporation of EEO-derived FA into milk fat. Cows were housed in individual tie-stalls at the UVM Paul Miller Research Facility, milked twice daily at 0400h and 1600h, and fed twice daily at 0600h and 1800h. Individual feed intakes were recorded and adjusted to achieve 10–15% refusals daily. Cows had continuous access to water. The four days prior to the start of the experiment served as the baseline period (control; **CON**), during which cows were fed the standard herd diet consisting of a mixed ration and top-dressed grain (Table 1). The two consecutive experimental periods were seven days each, and experimental diets were formulated for equal fat intake, consisting of 1.5% of DM as EEO plus 1.5% of DM as encapsulation matrix (Low-EEO; **LEO**) and 3% of DM as EEO (High-EEO; **HEO**). These percentages were applied to the DM intake (DMI) of the cows during the baseline period resulting in 380g each (190g at each feeding) of EEO plus encapsulation matrix being supplemented daily for the LEO treatment and 760g of EEO supplemented daily (380g at each feeding) for the HEO treatment. The supplements were thoroughly mixed with the top-dress grain and each cow was observed until the entirety of the supplement was consumed. EEO replaced an equal percentage of the top-dressed grain in the diets (Table 1). All diets were formulated to meet NRC 2001 requirements [11].

**Table 1:** Ingredient and nutrient composition of the diets.

	Treatment		
	CON^a^	LEO^b^	HEO^c^
Ingredient, % of DM			
Corn silage	28.3	28.3	28.3
3^rd^ cut mixed haylage	19.4	19.4	19.4
Concentrate^d^	37.2	37.2	37.2
Grain^e^	15.2	12.2	12.2
Lipid-encapsulated echium oil	—	1.5	3.0
Encapsulation matrix	—	1.5	—
Nutrient composition			
DM, %	41.0	40.6	48.0
CP^f^, % DM	15.2	14.4	14.2
aNDFom^g^, % DM	27.6	25.6	26.4
NFC^h^, % DM	38.7	38.7	38.6
NE_L_^i^, Mcal/kg	1.6	1.6	1.6
Fatty acids, % DM	3.0	5.5	5.3

^*a*^CON: control (0% of DM as encapsulated echium oil),

^*b*^LEO: 1.5% of DM as encapsulated echium oil, 1.5% DM as encapsulation matrix,

^*c*^HEO: 3% of DM as encapsulated echium oil.

^*d*^Concentrate contained (DM basis) 43.1% ground corn, 21.6% amino max, 16.2% citrus pulp, 8.6% canola meal, 5.4% soybean meal, 1.9% sodium sesquinate, 1.2% calcium carbonate, 1.0% salt, 0.5% magnesium oxide, 0.2% trace minerals, 0.1% vitamin mix, 0.1% Zinpro Availa^®^Plus, and 0.01% rumensin^®^.

^*e*^Grain contained (DM basis) 32.6% wheat midds, 20.0% steamed flaked corn, 16.1% soybean meal, 8.8% distiller’s grains, 6.5% fine corn meal, 4.8% heat-treated soy, 4% cane molasses, 2.2% calcium carbonate, 1.5% tallow, 1.4% bakery meal, 1.0% sodium sequicarbonate, 0.8% salt, 0.3% trace vitamins, 0.3% magnesium oxide.

^*f*^CP: Crude protein.

^*g*^aNDFom: Ash-corrected neutral detergent fiber.

^*h*^NFC: Non-fiber carbohydrates.

^*i*^NE_L_: Net energy lactation.

#### Data and Sample Collection

Milk weights were recorded and a 100 mL milk sample was taken at each milking. Milk samples were composited based on milk weight for each day and cow. An aliquot was preserved with 2-bromo-2-nitropropane-1,3-diol and analyzed for fat, protein, and lactose by Lancaster Dairy Herd Improvement Association (Manheim, PA). The cream layer was collected from a second aliquot by centrifugation at 3434 x *g* for 30 min at 8°C, and kept at -20°C until FA analysis. Feed and refusals were weighed and sampled daily. Both feed and refusal samples were composited per period for each cow, dried in a forced-air oven (VWR 1630, VWR, Radnor, PA) at 65°C for 48 h, and sent to Cumberland Valley Analytical Services Inc. (Hagerstown, MD) for chemical analysis of crude protein (CP) [12], neutral detergent fiber (NDF) [13], and ash [14]. Blood was collected into evacuated tubes containing K_2_EDTA (Becton Dickenson, Franklin Lakes, NJ) from the coccygeal vein at 0800 h (2 hours after feeding) on day -1 of the CON period and on day 2, 3, 4, and 7 of each experimental period. Blood samples were placed immediately on ice and plasma was obtained within 1 h of collection by centrifugation at 900 x *g* for 15 min at 4°C. Fecal matter was collected from each cow quantitatively for a 24 h period starting at 0830h on the last day of each period. Each fecal event was weighed, mixed thoroughly using an electric hand mixer, and subsampled. Fecal events were composited per cow and one aliquot was dried in a forced-air oven at 65°C for 48 h to determine DM. A second aliquot was lyophilized (FreeZone Plus 2.5, Labconco, Kansas City, MO) and stored at -20°C until subsequent FA analysis.

#### Forage, Fecal, Milk, and Plasma FA Analyses

Milk lipids were extracted using the method of Hara and Radin [15] and fatty acid methyl esters (FAME) were generated in a base-catalyzed transmethylation reported by Bainbrigde *et al*.[10]. Forage FAME were prepared as described by Sukhija and Palmquist [16] with the modifications by Bainbridge *et al*. [10] of using glyceryl tridecanoate (Nu-Check Prep, Elysian, MN, USA) as an internal standard (1 mg/mL in acetone). Plasma lipids were extracted using chloroform/methanol (2:1) as detailed by Folche *et al*. [17], isolated using solid-phase extraction, and methylated by the methods of Bainbridge *et al*. [10]. FAME from dried and ground forage samples, cream, and plasma were prepared and analyzed by gas-liquid chromatography (GLC) using the methods of Bainbridge *et al*.[10]. Fecal FAME were prepared and analyzed using the same procedure as for forage FA.

#### Statistical Analysis

Data were analyzed by repeated measures ANOVA using the PROC MIXED procedure in SAS (v. 9.4; SAS Institute, Cary, NC). The model included the fixed effect of diet, the fixed effect of period and sample day nested within period, the random effect of cow, and residual error. The interaction of period and diet was originally included in the model but removed because of *P*>0.15. For milk production and milk FA data, the last 4 days of each period were used to assess treatment effects. Differences between least-squares (LS) means were determined using the LSmeans/Diff option. Data were adjusted for multiple comparisons using Bonferroni’s method. Differences between FA intakes and fecal outputs were generated using the TTEST procedure with the PAIRED statement. Significance was declared at *P*<0.05 and trends at 0.05≤*P*<0.10.

## Results

### Experiment 1

**Fig 1:**
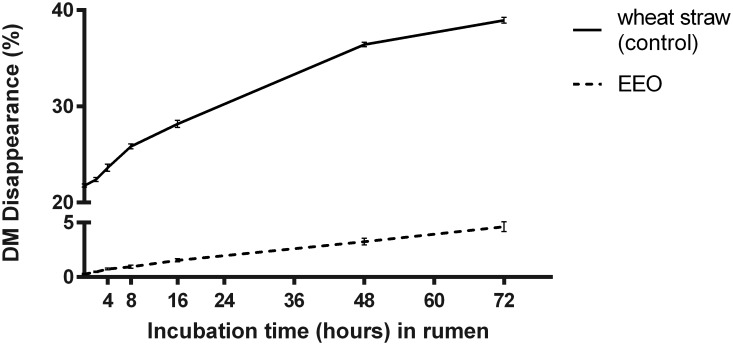
*In situ* ruminal dry matter (DM) disappearance (%) of EEO and wheat straw (control). Data are presented as LS means (n = 6) and standard error of the mean.

The EEO supplement lost 1.5% of DM after 16 h of rumen incubation and 3.3% DM after 48 h. Less than 5% DM disappearance was observed after 72 h of rumen incubation (Fig 1). The control (wheat straw) lost 28% of DM after 16 h and 39% after 72 h of incubation in the rumen.

### Experiment 2

#### Animal Intake and Performance

The encapsulation matrix consisted of exclusively SFA (Table 2) and hence, the EEO supplement was comprised of primarily SFA (66.6% of total FA) and only 17% of PUFA. Accordingly, the daily intake of SFA was highest on the LEO treatment, intermediate during HEO, and lowest during CON. The diets supplemented with either LEO or HEO provided more total FA than the CON diet (*P*<0.001, Table 3). Total PUFA, ALA, GLA, SDA, and total n-3 FA intakes increased in each experimental diet.

**Table 2:** Fatty acid composition (mg/g DM) of the diet components.

	Mixed-ration^a^	Grain	EEO^b^	Encapsulation Matrix
Total fatty acids	24.38	51.99	880.3	958.0
16:0	3.84	10.13	390.8	520.2
18:0	0.56	3.41	254.4	417.5
18:1 *c*9	5.85	12.89	39.61	—
18:2 *c*9,*c*12	10.20	21.99	36.05	—
18:3 *c*6,*c*9,*c*12	—	—	23.99	—
18:3 *c*9,*c*12,*c*15	2.22	1.53	81.56	—
18:4 *c*6,*c*9,*c*12,*c*15	—	—	31.14	—
∑ other^c^	1.70	2.03	16.54	20.31
Total SFA^d^	4.97	14.30	666.1	958.0
Total MUFA^e^	6.97	14.14	41.41	—
Total PUFA^f^	12.44	23.55	172.7	—
Total n-3	2.22	1.53	112.7	—
Total n-6	10.22	22.02	60.04	—
n-6/n-3 ratio	4.70	14.38	0.53	—

^*a*^The mixed-ration contained: 33.3% corn silage, 22.8% haylage, and 43.8% concentrate,

^*b*^EEO: encapsulated echium oil.

^*c*^Ʃ Other: 10:0, 12:0, 14:0, 16:1 *c*9, 16:1 *c*8, 16:1 *c*11, 18:1 *c*11, 20:0, 20:1 *c*11, 20:2 *c*11,*c*14, 22:0, 22:1 *c*13, 20:4 *c*5,*c*8,*c*11,*c*14, 24:0, 24:1 *c*15.

^*d*^Total SFA: sum of saturated fatty acids (4:0 to 26:0).

^*e*^Total MUFA: sum of monounsaturated fatty acids (14:1 to 24:1).

^*f*^Total PUFA: sum of polyunsaturated fatty acids (18:2 to 22:5).

**Table 3:** Dry matter (kg/day) and fatty acid intake (g/day) of dairy cows^a^ on CON^b^, LEO^c^, and HEO^d^ diets.

	Treatment			SE	*P*-value
	CON^b^	LEO^c^	HEO^d^		
Dry matter intake, kg/d	27.3	28.0	30.4	1.6	0.09
Total fatty acids	808.1a	1538.6b	1596.9b	68.20	<0.001
16:0	132.6a	508.1b	486.2b	21.26	<0.001
18:0	26.94a	312.4c	260.4b	12.07	0.003
18:1 *c*9	197.0a	202.5a	229.3b	9.85	0.016
18:2 *c*9,*c*12	332.7	341.2	372.9	16.32	0.071
18:3 *c*9,*c*12,*c*15	68.77a	84.53b	133.2c	4.96	<0.001
18:3 *c*6,*c*9,*c*12	0.00a	10.29b	21.38c	0.72	<0.001
18:4 *c*6,*c*9,*c*12,*c*15	0.00a	13.33b	27.79c	0.94	<0.001
Ʃ other^e^	50.18a	62.40b	64.96b	2.85	0.003
Total SFA^f^	176.6a	854.5c	778.5b	34.61	<0.001
Total MUFA^g^	229.6a	234.2a	262.7b	11.35	0.029
Total PUFA^h^	401.9a	449.9b	555.7c	22.76	<0.001
Total n-3	68.77a	97.87b	161.0c	5.88	<0.001
Total n-6	333.2a	352.0a	394.8b	16.99	0.013

^*a*^LS means are based on 6 dairy cows per treatment.

^*b*^CON: control (0% of DM as encapsulated echium oil).

^*c*^LEO: 1.5% of DM as encapsulated echium oil, and 1.5% of DM as encapsulation matrix,

^*d*^HEO: 3% of DM as encapsulated echium oil.

^*e*^Ʃ Other: 10:0, 12:0, 14:0, 16:1 *c*9, 16:1 *c*8, 16:1 *c*11, 18:1 *c*11, 20:0, 20:1 *c*11, 20:2 *c*11,*c*14, 22:0, 22:1 *c*13, 20:4 *c*5,*c*8,*c*11,*c*14, 24:0, 24:1 *c*15.

^*f*^Total SFA: sum of saturated fatty acids (4:0 to 26:0).

^*g*^Total MUFA: sum of monounsaturated fatty acids (14:1 to 24:1).

^*h*^Total PUFA: sum of polyunsaturated fatty acids (18:2 to 22:5).

The inclusion of EEO in the diet at 1.5% and 3% of DM did not affect milk components (S1 Table). DMI tended to be higher on the HEO treatment when compared to the CON treatment (30.4 *vs*. 27.3 kg/day, respectively; *P* = 0.09, Table 3). This coincided with a trend of increased total fecal weight during the HEO treatment when compared to CON (8.6 *vs*. 7.4 kg DM/day, respectively; *P* = 0.06). Milk production tended to be higher in the LEO and HEO treatments when compared to CON (44.8 *vs*. 41.4 kg/day, respectively; *P* = 0.07).

#### Milk FA Profile

The milk fat content of total SFA was lower during the HEO treatment (68.2 g/100g FA) when compared to CON (69.6 g/100g FA; *P* = 0.007; Table 4), while total PUFA were higher during HEO than during CON and LEO (4.52 vs. 3.96, and 4.06 g/100g FA, respectively; *P*<0.001). Total milk n-3 FA increased with increasing EEO supplementation (0.41, 0.59, 0.77 g/100g FA for CON, LEO, and HEO, respectively; *P*<0.001). This was driven by the increase of ALA in milk fat with each addition of EEO to the diet (0.33, 0.47, 0.59 g/100g for CON, LEO, and HEO, respectively; *P*<0.001). SDA increased in response to EEO supplementation from undetectable in CON to 0.05 and 0.08 g/100g FA during the LEO and HEO treatments, respectively (*P*<0.001). The milk fat content of eicosapentaenoic acid (20:5 *c*5,*c*8,*c*11,*c*14,*c*17; EPA) was higher in HEO-fed cows than in CON (0.04 vs. 0.03 g/100g FA; *P*<0.001). GLA increased with increasing EEO supplementation (0.02, 0.04, 0.07 g/100g FA, respectively; *P*<0.001). Milk fat of HEO-fed cows contained a higher content of *trans*-18:1 FA than LEO- and CON-fed cows (2.56 vs. 2.03 and 2.23 g/100g FA, respectively; *P*<0.001). Rumenic acid (18:2 *c*9, *t*11), the predominant CLA isomer, was highest in HEO (0.58 vs. 0.52, and 0.44 for HEO, LEO, and CON, respectively; *P*<0.001).

**Table 4:** Content of selected fatty acids (g/100g FA) in milk fat of dairy cows^a^ on CON^b^, LEO^c^, and HEO^d^ diets.

Fatty acid	Treatment			SE	*P-*value
	CON	LEO	HEO		
16:0	32.66a	33.19a	31.47b	0.77	0.009
18:0	9.52ab	8.92b	9.80a	0.27	0.025
18:1 *t*11	0.95a	1.04a	1.23b	0.08	<0.001
18:1 *c*9	17.67	17.53	17.58	0.71	ns
18:2 *c*9,*c*12	1.96	1.95	2.06	0.10	ns
18:3 *c*6,*c*9,*c*12	0.02a	0.04b	0.07c	0.00	<0.001
18:3 *c*9,*c*12,*c*15	0.33a	0.47b	0.59c	0.02	<0.001
18:2 *c*9,*t*11	0.44b	0.52b	0.58a	0.03	<0.001
18:4 *c*6,*c*9,*c*12,*c*15	0.00c	0.05b	0.08a	0.00	<0.001
20:4 *c*5,*c*8,*c*11,*c*14	0.13	0.13	0.12	0.00	ns
20:5 *c*5,*c*8,*c*11,*c*14,*c*17	0.03b	0.03b	0.04a	0.00	<0.001
22:5 *c*7,*c*10,*c*13,*c*16,*c*19	0.04	0.04	0.04	0.00	ns
*de novo*^e^	27.58	27.35	27.03	0.39	ns
Mixed^f^	34.98a	35.47a	33.54b	0.85	0.003
Preformed^g^	35.07b	34.99b	37.05a	1.11	0.004
Total SFA^h^	69.55a	69.12ab	68.18b	0.93	0.007
Total MUFA^i^	24.62	24.88	25.14	0.76	ns
Total PUFA^j^	3.69b	4.06b	4.52a	0.17	<0.001
Total n-6^k^	2.30	2.30	2.37	0.09	ns
Total n-3^l^	0.41c	0.59b	0.77a	0.02	<0.001
n-6:n-3 ratio	5.63a	3.85b	3.10c	0.03	<0.001
Total *trans* 18:1	2.03b	2.23b	2.56a	0.14	<0.001
Total CLA^m^	0.48b	0.55ab	0.61a	0.04	<0.001
Total odd and branched-chain FA	1.62a	1.48b	1.49b	0.04	0.003

^*a*^LS means are based on 6 dairy cows per treatment. LS means without a common letter differ significantly (*P*<0.05).

^*b*^CON: control (0% of DM as encapsulated echium oil).

^*c*^LEO: 1.5% of DM as encapsulated echium oil, and 1.5% of DM as encapsulation matrix,

^*d*^HEO: 3% of DM as encapsulated echium oil.

^*e*^*de novo*: sum of fatty acids <16 carbons.

^*f*^Mixed: sum of 16:0 and 16:1 *c*9.

^*g*^Preformed: sum of fatty acids >16 carbons.

^*h*^Total SFA: sum of saturated fatty acids (4:0 to 26:0).

^*i*^Total MUFA: sum of monounsaturated fatty acids (14:1 to 24:1).

^*j*^Total PUFA: sum of polyunsaturated fatty acids (18:2 to 22:5).

^*k*^Total n-6: sum of n-6 PUFA: 18:2 *c*9,*c*12, 18:3 *c*6,*c*9,*c*12, 20:2 *c*11,*c*14, 20:3 *c*8,*c*11,*c*14, 20:4 *c*5,*c*8,*c*11,*c*14, and 22:4 *c*7,*c*10,*c*13,*c*16.

^*l*^Total n-3: sum of n-3 PUFA: 18:3 *c*9,*c*12,*c*15, 18:4 *c*6,*c*9,*c*12,*c*15, 20:3 *c*11,*c*14,*c*17, 20:4 *c*8,*c*11,*c*14,*c*17, 20:5 *c*5,*c*8,*c*11,*c*14,*c*17, and 22:5 *c*7,*c*10,*c*13,*c*16,*c*19.

^*m*^Total CLA: sum of conjugated linoleic acid isomers: 18:2 *c*9,*t*11, 18:2 *c*9,*c*11, 18:2 *t*7,*t*9, and 18:2 *t*10,*t*12.

#### Temporal Incorporation of FA into Milk and Plasma

**Fig 2:**
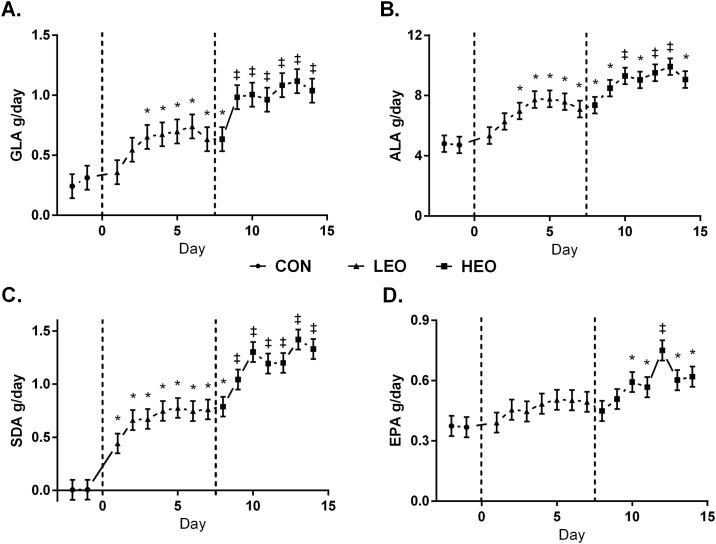
Temporal pattern of milk fatty acid yield (g/day) of γ-linoleic acid (GLA) [A], α-linolenic acid (ALA) [B], steariodonic acid (SDA) [C], and eicosapentaenoic acid (EPA) [D] from cows on baseline diet (CON: ●), supplemented with 1.5% of DM as encapsulated echium oil (EEO) and 1.5% of DM as encapsulation matrix (LEO:▲), and 3% DM as EEO (HEO:■). Data are presented as LSmeans (n = 6) and standard error. * = days significantly different from CON, ‡ = days significantly different from CON and LEO (*P*<0.05).

**Fig 3:**
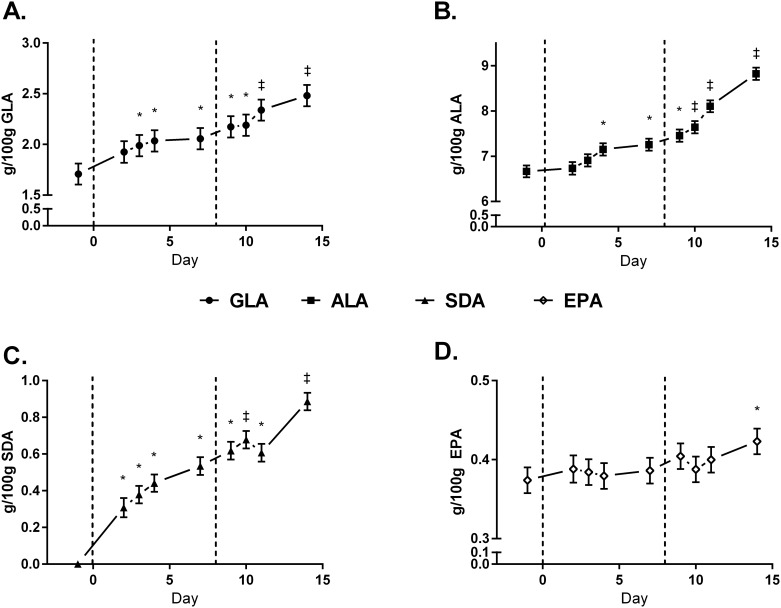
Temporal incorporation (g/100g) of γ-linolenic acid (GLA) [A], α-linolenic acid (ALA) [B], steariodonic acid (SDA) [C], and eicosapentaenoic acid (EPA) [D] into plasma cholesterol esters of cows on baseline diet (CON), supplemented with 1.5% of DM as encapsulated echium oil (EEO) and 1.5% of DM as encapsulation matrix (LEO), and 3% DM as EEO (HEO). Data are presented as LSmeans (n = 6) and standard error. * = days significantly different from CON, ‡ = days significantly different from CON and LEO (*P*<0.05).

**Fig 4:**
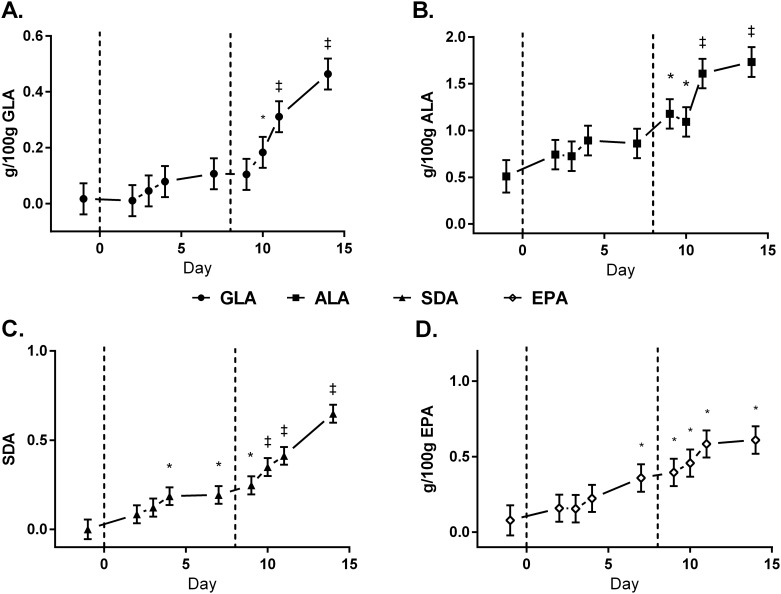
Temporal incorporation (g/100g) of γ-linolenic acid (GLA) [A], α-linolenic acid (ALA) [B], steariodonic acid (SDA) [C], and eicosapentaenoic acid (EPA) [D] in plasma free fatty acids of cows on baseline diet (CON), supplemented with 1.5% of DM as encapsulated echium oil (EEO) and 1.5% of DM as encapsulation matrix (LEO), and 3% DM as EEO (HEO). Data are presented as LSmeans (n = 6) and standard error. * = days significantly different from CON, ‡ = days significantly different from CON and LEO (*P*<0.05).

**Fig 5:**
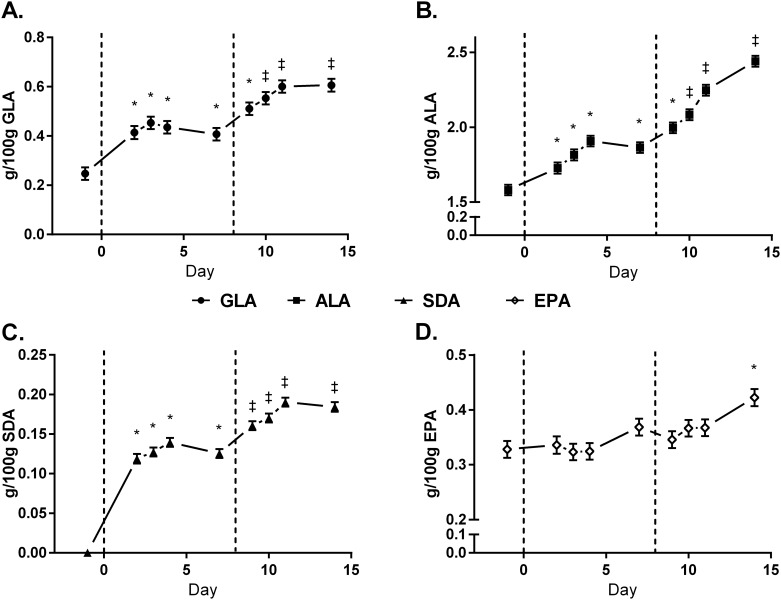
Temporal incorporation (g/100g) of γ-linolenic acid (GLA) [A], α-linolenic acid (ALA) [B], steariodonic acid (SDA) [C], and eicosapentaenoic acid (EPA) [D] in plasma phospholipids of cows on baseline diet (CON), supplemented with 1.5% of DM as encapsulated echium oil (EEO) and 1.5% of DM as encapsulation matrix (LEO), and 3% DM as EEO (HEO). Data are presented as LSmeans (n = 6) and standard error. * = days significantly different from CON, ‡ = days significantly different from CON and LEO (*P*<0.05).

**Fig 6:**
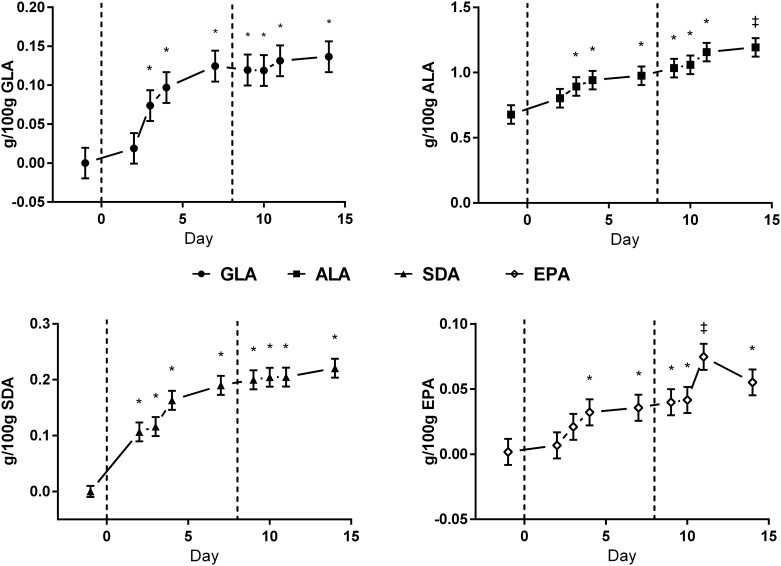
Temporal incorporation (g/100g) of γ-linolenic acid (GLA) [A], α-linolenic acid (ALA) [B], steariodonic acid (SDA) [C], and eicosapentaenoic acid (EPA) [D] in plasma triacylglycerides of cows on baseline diet (CON), supplemented with 1.5% of DM as encapsulated echium oil (EEO) and 1.5% of DM as encapsulation matrix (LEO), and 3% DM as EEO (HEO). Data are presented as LSmeans (n = 6) and standard error. * = days significantly different from CON, ‡ = days significantly different from CON and LEO (*P*<0.05).

The temporal incorporation of EO-derived FA into milk fat is presented in Fig 2. There were no differences in the transfer efficiencies of EEO-derived FA into milk fat between LEO and HEO treatments (Table 5). The temporal incorporation of GLA, ALA, SDA, and EPA into plasma cholesterol esters (CE), free fatty acids (FFA), phospholipids (PL), and triacylglycerols (TAG) is presented in Figs 3–6. Full FA profiles of all plasma lipid fractions are available in S3–S6 Tables.

**Table 5:** Transfer efficiencies^a^ (%) of fatty acids derived from encapsulated-echium oil into milk fat.

	Treatment		SE	*P*-Value
	LEO^b^	HEO^c^		
18:3 *c*6,*c*9,*c*12	4.74	4.36	0.86	ns
18:3 *c*9,*c*12,*c*15	9.01	7.46	0.78	ns
18:4 *c*6,*c*9,*c*12,*c*15	5.92	5.19	0.48	ns
18:4 *c*6,*c*9,*c*12,*c*15 plus 20:5 *c*5,*c*8,*c*11,*c*14,*c*17^d^	6.98	6.30	0.65	ns

^*a*^LS means are based on 6 dairy cows per treatment.

^*b*^LEO: 1.5% of DM as encapsulated echium oil, and 1.5% of DM as encapsulation.

^*c*^HEO: 3% of DM as encapsulated echium oil.

^*d*^18:4 c6,c9,c12,c15 (SDA) plus 20:5 c5,c8,c11,c14,c17 (EPA): [((SDA + EPA (g/day) in milk fat of treatment)–(SDA + EPA (g/day) in milk fat of control))/ SDA (g/day) in diet]*100.

#### Fecal FA Output

**Fig 7:**
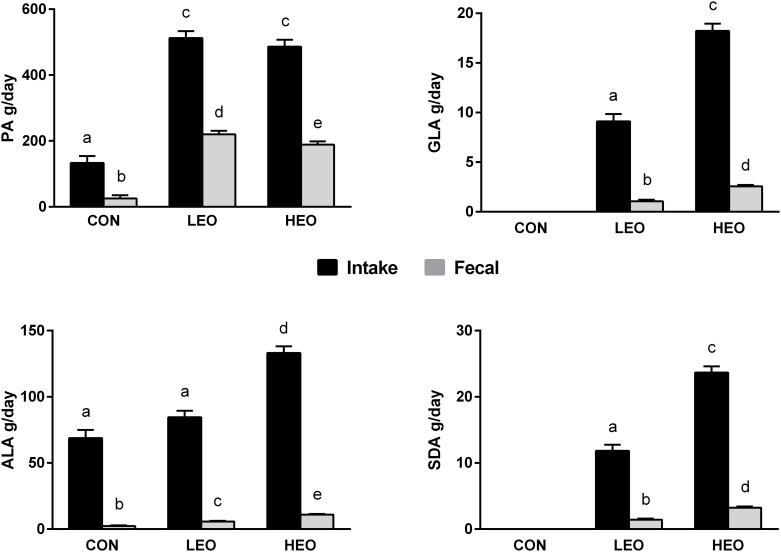
Fecal output of EEO-derived FA; palmitic acid (PA) [A], γ-linolenic acid (GLA) [B], α-linolenic acid (ALA) [C], steariodonic acid (SDA) [D] in comparison to intake of cows on baseline diet (CON), supplemented with 1.5% of DM as encapsulated echium oil (EEO) and 1.5% of DM as encapsulation matrix (LEO), and 3% DM as EEO (HEO). LSmeans (n = 6) and standard error. Means without a common letter differ significantly, *P*<0.05.

The EEO and encapsulation matrix were visually evident in the feces (S1 Fig). Palmitic acid (16:0; PA) made up 52% of the encapsulation matrix, this FA was excreted in the feces at a rate of 43% and 39% on the LEO and HEO treatments, respectively, vs. 19% on CON (*P*<0.001; Fig 7). Since ALA was not solely derived from the EEO supplement, we only accounted for ALA derived from EEO and normalized for the amount of ALA excreted in feces during CON. LEO-fed cows excreted 7% of EEO-derived ALA while HEO-fed cows excreted 8% of EEO-derived ALA. For both GLA and SDA, 12% and 14% of EEO-derived FA remained unabsorbed by LEO- and HEO-fed cows.

## Discussion

The objectives of this study were to evaluate the ruminal protection and apparent total-tract digestibility of echium oil, using lipid-encapsulation as the encapsulate technology, and the incorporation of EO-derived FA into plasma lipid fractions and milk fat. Rumen-protection of UFA by lipid encapsulation with hydrogenated oil makes the UFA unavailable to bacterial enzymes by increasing the melting point of the fat supplement, while decreasing solubility. Hence, the calculation of DM disappearance using the *in situ* nylon bag technique assumes that physical losses are due to the dissociation of FA from the supplement, and that biohydrogenation of non-dissociated FA is minimal. To the authors’ knowledge, no study has evaluated rumen-stability of lipid-encapsulated oils, although a lipid-encapsulated lysine product showed rumen stability at 92–95% of DM after 24 h [18]. The negligible loss of DM from the EEO supplement after 72 h of rumen incubation suggests that the product is rumen stable, as average retention time within the rumen is 8–30 hours depending on particle size, with smaller particles moving more rapidly through the rumen than larger particles [19].

### Milk FA Composition

The milk FA profile was altered following the inclusion of EEO in the diet, as demonstrated previously [10], and contents of ALA, SDA, EPA, total n-3, and GLA were similar among studies. The time-course incorporation of EO-derived FA and their downstream metabolites into milk fat was evaluated in this study, and, in accordance with other studies, incorporation of dietary FA occurred within 2–5 days [20–22]. Incorporation of EPA, however, was slower, potentially due to the 25% bioconversion rate of SDA to EPA [23]. Bernal-Santos *et al*.[21] demonstrated that EPA levels in milk plateaued after day 3 of ruminal infusion of SDA-rich soybean oil, however, if treatments in the current study had been longer, EPA levels in milk and plasma may have increased.

Total *trans* 18:1 and CLA were increased in milk fat of the HEO treatment suggesting some EEO-derived PUFA were released from the supplement and biohydrogenated, which could explain, in part, the lower transfer efficiency of ALA, SDA, and GLA into milk fat. However, these increases were negligible, and the increase in biohydrogenation intermediates in the HEO vs. LEO treatment did not produce a difference in transfer efficiencies between the treatments. In the current study, transfer efficiencies were higher than previously shown [10]. This could be due to the method of supplementation (top dressed vs. tumble-mixed) as the current study did not allow for EEO in refusals. Overall, the transfer efficiencies of ALA, SDA, and GLA in the current study are comparable to other research using rumen-protected flax oil[24] or above the transfer efficiencies for EPA and docosahexaenoic acid (DHA) described by Chillard *et al*.[25].

### Temporal Incorporation of EO-Derived FA into Plasma Lipid Fractions

When cows are in positive energy balance, only small amounts of PUFA are used for energy; instead, these FA are protected from degradation through preferential incorporation into plasma PL and CE [26,27], as validated in the current study. n-3 FA are preferentially transported in plasma PL and CE so that they can be used as precursors for signaling molecules such as prostaglandins, leukotrienes, eicosanoids, and thromboxanes. Stamey *et al*.[22] also observed a preferential incorporation of the n-3 FA, DHA, into plasma PL when supplementing dairy cows with rumen-protected algal oil and algal biomass for 7 days.

Plasma TAG are the primary source of FA to the mammary gland for milk fat synthesis, whereas plasma FFA are available for direct uptake, and the utilization of FFA is dependent on their plasma concentration [28]. Incorporation of GLA, ALA, and SDA into plasma TAG and milk fat both occurred by day 3, demonstrating that a portion of these FA were readily available in the small intestine. It is noteworthy that content of EPA in plasma TAG was increased on day 4, yet, higher contents in milk fat were not observed until day 9. This finding supports the hypothesis by Stamey *et al*.[29] that the action of lipoprotein lipase in the mammary gland is dependent on the FA composition of chylomicrons. GLA, ALA, and SDA in plasma TAG did not increase with the additional intake of EEO from the LEO to HEO treatment. This suggests that i) increased supplementation did not result in increased bioavailability in the small intestine, ii) the metabolism and/or catabolism of these FA increased, or iii) incorporation into tissues and/or removal from plasma increased with the additional intake of EEO.

### Apparent Total-Tract Digestibility of EEO

While consuming the LEO- and HEO-supplemented diets, cows excreted more than two-fold ALA (6.7 and 8.2%, respectively) when compared to CON (3.2%), suggesting that the lipid encapsulation matrix (hydrogenated vegetable oil) likely inhibited the digestibility of PUFA. SFA have been shown to be less digestible than unsaturated fats [30] and PA excretion in feces was two-fold higher on LEO and HEO treatments (43 and 39%, respectively) in comparison to CON (19%). These data are corroborated by the visual evidence of the EEO supplement and encapsulation matrix in the feces of the dairy cows, indicating that a large proportion of the supplement passed through the cow unchanged. Pappritz *et al*.[31] examined the digestibility of CLA encapsulated in hydrogenated fat fed at 50 and 100 g/day and demonstrated a similar excretion of PA (39.5% and 42.8%, respectively) compared to the current study. Digestibility may also be reduced in particular with hydrogenated fat supplements which have a higher melting point and lower solubility than UFA. A meta-analysis of FA digestibility in ruminants showed that inclusion of hydrogenated tallow decreases the intestinal absorption of FA by 23–53% [32]. Moreover, Weiss and Wyatt [33] demonstrated a decreased digestibility of a hydrogenated fat source (38.1% digestibility) when compared to calcium salts of palm FA (87.5% digestibility). Lastly, silica was used as a binder in the encapsulation matrix and hence occurred in the EEO supplement. This could have affected the absorption of EO-derived FA as forages containing silica have been shown to decrease FA digestibly [34].

Once released, the EO-derived FA may also undergo biohydrogenation in the large intestine, leading to heightened fecal output of biohydrogenation intermediates [35]. The increased proportions of the biohydrogenation intermediates, such as CLA and *trans*-18:1 isomers, as well as the biohydrogenation end product 18:0, in fecal matter during the LEO and HEO treatments suggests that EEO-derived PUFA became partly available in the animals’ hindgut resulting in microbial biohydrogenation processes. Demeyer *et al*.[36] demonstrated the site of microbial FA modification shifts to the large intestine when high-fat diets are fed. Similar results were observed by Côrtes *et al*.[37] who supplemented 19 g/kg DM as calcium salts of flaxseed oil, resulting in an increased proportion of fecal 18:0, causing a negative apparent total-tract digestibility. Fecal CLA and *trans*-18:1 FA, however, were not reported. The time-course adaption of hindgut bacteria to lipid supplementation is unknown, thus the authors acknowledge that the short periods in the current study may not fully account for the shifting bacterial populations in the hindgut.

During the HEO treatment, 5% of SDA was incorporated into milk fat and 14% was excreted in the feces, resulting in 81% of dietary SDA being unaccounted for. This rather large proportion of SDA may have been i) incorporated into tissues, which is supported by our observation of increased SDA in plasma PL and CE, ii) biohydrogenatated by rumen and/or hindgut microbes, or iii) metabolized (*i*.*e*., elongation, desaturation, or β-oxidation). The increase in CLA and *trans*-18:1 in milk, plasma lipids, and feces of HEO-fed cows suggests some SDA may have been biohydrogenated[38]. However, the net biohydrogenation of SDA cannot be estimated, as biohydrogenation of SDA follows the same pathway of other 18-carbon UFA[39]. SDA that was metabolized into EPA and incorporated into milk accounts for an additional 1% of dietary SDA, and fecal excretion of EPA accounts for an additional 3%. EPA was increased in all plasma lipid fractions, yet, this study could not assess the percentage of dietary SDA that was converted into EPA and transported in plasma, nor the amount of dietary SDA that was subject to β-oxidation or converted into signaling molecules. Research shows that transfer efficiencies of SDA into milk fat can reach as high as 47% (accounting for down-stream metabolites) when this FA is infused into the abomasum [21]. This higher transfer efficiency of PUFA into milk fat in comparison to the current rumen-protection technologies suggests future research should focus on improving rumen-protection methods to achieve optimal transfer efficiencies of PUFA into milk fat.

### Conclusion

The successful protection of PUFA from biohydrogenation in the rumen is difficult to achieve, with most rumen-protection technologies allowing for some modification of PUFA in the rumen. Moreover, if a product is rumen stable, it must subsequently release the FA for absorption in the small intestine. This study has demonstrated that the same technology that imparts rumen-protection also significantly inhibits the FA availability for intestinal absorption, resulting in large proportions being excreted into the feces. In addition, EEO-derived PUFA were not incorporated in sufficient proportions into plasma lipid fractions available to the mammary gland, leading to an overall low transfer efficiency of EEO-derived PUFA into milk fat. Overall, lipid-encapsulation appears to be an inadequate rumen-protection method to provide optimal transfer of PUFA into milk fat.

## Supporting Information

S1 Fig Visual evidence of encapsulated echium oil (EEO) and encapsulation matrix [A] in feces of cows on baseline diet [B], supplemented with 1.5% of DM as EEO and 1.5% of DM as encapsulation matrix [C], and 3% DM as EEO [D]. (TIF)

S1 Table Daily dry matter intake (DMI), milk yield, milk components, and feed efficiency of dairy cows on CON, LEO, and HEO diets. (DOCX)

S2 Table Fatty acid composition (g/100g FA) of milk fat by day in response to CON, LEO, and HEO diets. (DOCX)

S3 Table Fatty acid composition (g/100g FA) of the cholesterol ester plasma lipid fraction by day in response to CON, LEO, and HEO diets. (DOCX)

S4 Table Fatty acid composition (g/100g FA) of the free fatty acid plasma lipid fraction by day in response to CON, LEO, and HEO diets. (DOCX)

S5 Table Fatty acid composition (g/100g FA) of the phospholipid plasma lipid fraction by day in response to CON, LEO, and HEO diets. (DOCX)

S6 Table Fatty acid composition (g/100g FA) of the triacylglycerol plasma lipid fraction by day in response to CON, LEO, and HEO diets. (DOCX)

S7 Table Fatty acid content of fecal matter (mg/g DM) from dairy cows on CON, LEO, and HEO diets. (DOCX)

## References

[1] American Heart Association. Dietary guidelines advisory. 2015.

[2] MacGibbon AKH, Taylor MW. Composition and structure of bovine milk lipids In: Fox P, McSweeney P, editors. Advanced Dairy Chemistry Volume 2 Lipids. New York, USA: Springer; 2006 pp. 1–42.

[3] Harfoot C, Hazlewood G. Lipid metabolism in the rumen In: Stewart C, Hobson P, editors. The Rumen Microbial Ecosystem. London, UK: Blackie; 1997 pp. 382–426.

[4] Jenkins TC. Lipid metabolism in the rumen. J Dairy Sci. 1993;76: 3851–3863. 10.3168/jds.S0022-0302(93)77727-9 8132891

[5] Palmquist DL. Biohydrogenation then and now. Eur J Lipid Sci Technol. 2007;109: 737–739. 10.1002/ejlt.200700146

[6] Bauman DE, Griinari JM. Regulation and nutritional manipulation of milk fat: Low-fat milk syndrome. Livest Prod Sci. 2001;70: 15–29. 10.1016/S0301-6226(01)00195-6 10959429

[7] Palmquist DL, Beaulieu AD, Barbano DM. Feed and animal factors influencing milk fat composition. J Dairy Sci. 1993;76: 1753–1771. 8326036 10.3168/jds.s0022-0302(93)77508-6

[8] Jenkins TC, Bridges WC. Protection of fatty acids against ruminal biohydrogenation in cattle. Eur J Lipid Sci Technol. 2007;109: 778–789. 10.1002/ejlt.200700022

[9] Kliem KE, Shingfield KJ. Manipulation of milk fatty acid composition in lactating cows: Opportunities and challenges.: 1–68.

[10] Bainbridge ML, Lock AL, Kraft J. Lipid-encapsulated echium oil (*Echium plantagineum*) increases the content of stearidonic acid in plasma lipid fractions and milk fat of dairy cows. J Agric Food Chem. 2015;63: 4827–4835. 10.1021/acs.jafc.5b00857 25904162

[11] Nutrition requiremnets for dairy cattle. 7th revise Washington D.C.: Subcommitee on Dairy Cattle Nutrition; 2001.

[12] Association of Official Analytical Chemists. Protein (Crude) in Animal Feed (990.03) Official Methods of Analysis, 17th edition Gaithersburg, MD, USA; 2000.

[13] Van Soest PJ, Robertson JB, Lewis BA. Methods for dietary fiber, neutral detergent fiber, and nonstarch polysaccharides in relation to animal nutrition. J Dairy Sci. Elsevier; 1991;74: 3583–3597. 10.3168/jds.S0022-0302(91)78551-2 1660498

[14] Association of Official Analytical Chemists. Ash of Animal Feed (942.05) Official Methods of Analysis, 17th edition 2000.

[15] Hara A, Radin NS. Lipid extraction of tissues with a low-toxicity solvent. Anal Biochem. 1978;90: 420–6. 727482 10.1016/0003-2697(78)90046-5

[16] Sukhija PS, Palmquist DL. Rapid method for determination of total fatty acid content and composition of feedstuffs and feces. J Agric Food Chem. 1988;36: 1202–1206. 10.1021/jf00084a019

[17] Folch J, Lees M, Stanley GHS. A Simple Method for the Isolation and Purification of Total Lipides from Animal Tissues. J Biol Chem. 1957;226: 497–509. 13428781

[18] Gressley TF, de Veth MJ, Diana NK, Mackey E. Evaluating in situ procedures for testing lipid encapsulated products—lysine as an example. J Dairy Sci. 2012;95: 353.22192214

[19] Nobel RC. Digestion, absorption, and transport of lipids in ruminant animals. Prog Lipid Res. 1978;17: 55–91. 34168 10.1016/0079-6832(78)90005-8

[20] Kitessa S, Young P. Enriching milk fat with n−3 polyunsaturated fatty acids by supplementing grazing dairy cows with ruminally protected Echium oil. Anim Feed Sci Technol. 2011;170: 35–44.

[21] Bernal-Santos G, O’Donnell a M, Vicini JL, Hartnell GF, Bauman DE. Hot topic: Enhancing omega-3 fatty acids in milk fat of dairy cows by using stearidonic acid-enriched soybean oil from genetically modified soybeans. J Dairy Sci. Elsevier; 2010;93: 32–7. 10.3168/jds.2009-2711 20059901

[22] Stamey J., Shepherd D., de Veth M., Corl B. Use of algae or algal oil rich in n-3 fatty acids as a feed supplement for dairy cattle. J Dairy Sci. Elsevier; 2012;95: 5269–75. 10.3168/jds.2012-5412 22916931

[23] Whelan J. Dietary Stearidonic Acid Is a Long Chain (n-3) Polyunsaturated Fatty Acid with Potential Health Benefits. J Nutr. 2009;139: 5–10. 10.3945/jn.108.094268 19056654

[24] Hawkins A, Yuan K, Armendariz C, Highland G, Bello N, Winowiski T, et al Effects of urea formaldehyde condensation polymer treatment of flaxseed on ruminal digestion and lactation in dairy cows. J Dairy Sci. Elsevier; 2013;96: 3907–15. 10.3168/jds.2012-6207 23548281

[25] Chilliard Y, Ferlay A, Doreau M. Effect of different types of forages, animal fat or marine oils in cow’s diet on milk fat secretion and composition, especially conjugated linoleic acid (CLA) and polyunsaturated fatty acids. Livest Prod Sci. 2001;70: 31–48.

[26] Brumby PE, Storry JE, Sutton JD. Metabolism of cod-liver oil in relation to milk fat secretion. J Dairy Res. 1972;39: 167–182. 5044765 10.1017/s0022029900013960

[27] Offer NW, Speake BK, Dixon J, Marsden M. Effect of fish-oil supplementation on levels of (n-3) poly-unsaturated fatty acids in the lipoprotein fractions of bovine plasma. Anim Sci. 2001;73: 523–531.

[28] Palmquist DL. Milk Fat: Origin of Fatty Acids and Influence of Nutritional Factors Thereon Advanced Dairy Chemistry, Vol 2 2006 pp. 43–93.

[29] Stamey Lanier J, Suagee JK, Becvar O, Corl B. Mammary uptake of fatty acids supplied by intravenous triacylglycerol infusion to lactating dairy cows. Lipids. 2013;48: 469–79. 10.1007/s11745-013-3782-6 23504269

[30] Firkins JL, Eastridge ML. Assessment of the effects of iodine value on fatty acid digestibility, feed intake, and milk production. J Dairy Sci. 1994;77: 2357–2366. 10.3168/jds.S0022-0302(94)77178-2 7962857

[31] Pappritz J, Lebzien P, Meyer U, Jahreis G, Kramer R, Flachowsky G, et al Duodenal availability of conjugated linoleic acids after supplementation to dairy cow diets. Eur J Lipid Sci Technol. 2011;113: 1443–1455. 10.1002/ejlt.201100170

[32] Glasser F, Schmidely P, Sauvant D, Doreau M. Digestion of fatty acids in ruminants: a meta-analysis of flows and variation factors: 2. C18 fatty acids. Animal. 2008;2: 691–704. 10.1017/S1751731108002036 22443594

[33] Weiss WP, Wyatt DJ. Digestible energy values of diets with different fat supplements when fed to lactating dairy cows. J Dairy Sci. Elsevier; 2004;87: 1446–1454. 10.3168/jds.S0022-0302(04)73295-6 15290993

[34] Van Soest PJ, Jones LHP. Effect of Silica in Forages upon Digestibility. J Dairy Sci. 1968;51: 1644–1648. 10.3168/jds.S0022-0302(68)87246-7

[35] Pantoja J, Firkins JL, Eastridge ML. Fatty Acid Digestibility and Lactation Performance by Dairy Cows Fed Fats Varying in Degree of Saturation. J Dairy Sci. 1996;79: 429–437. 10.3168/jds.S0022-0302(96)76382-8 8708104

[36] Demeyer DI. Quantitative aspects of microbial metabolism in the rumen and hindgut In: Jouany JP, editor. Rumen Microbial Metabolism and Ruminant Digestion. INRA Editi Paris, France; 1991 pp. 217–237.

[37] Côrtes C, da Silva-Kazama DC, Kazama R, Gagnon N, Benchaar C, Santos GTD, et al Milk composition, milk fatty acid profile, digestion, and ruminal fermentation in dairy cows fed whole flaxseed and calcium salts of flaxseed oil. J Dairy Sci. Elsevier; 2010;93: 3146–57. 10.3168/jds.2009-2905 20630232

[38] Maia MRG, Correia CAS, Alves SP, Fonseca AJM, Cabrita ARJ. Technical note: Stearidonic acid metabolism by mixed ruminal microorganisms in vitro. J Anim Sci. 2012;90: 900–904. 10.2527/jas.2011-4118 22021809

[39] Jalc D, Certik M, Kundrikova K, Namestkova P. Effect of unsaturated C18 fatty acids (oleic, linoleic and alpha-linolenic acid) on ruminal fermentation and production of fatty acid isomers in an artificial rumen. Vet Med (Praha). 2007;52: 87–94.

