# Supplementary material for: Lipid Encapsulation Provides Insufficient Total-Tract Digestibility to Achieve an Optimal Transfer Efficiency of Fatty Acids to Milk Fat
Source: PLoS One. 2016 Oct 14;11(10):e0164700. doi: 10.1371/journal.pone.0164700 (PMC5065208; doi:10.1371/journal.pone.0164700)
Supplement: S1 Table — (DOCX) [file pone.0164700.s002.docx]

**Supplemental Table 1.** Daily dry matter intake (DMI), milk yield, milk components, and feed efficiency of dairy cows*^a^* on CON*^b^*, LEO*^c^*, and HEO*^d^* diets.

|  | **Treatment** | | | **SE** | ***P*-value** |  |
| --- | --- | --- | --- | --- | --- | --- |
|  | **CON** | **LEO** | **HEO** |  |  |  |
| DMI, kg/d | 27.3 | 28.0 | 30.4 | 1.6 | 0.09 |  |
|  |  |  |  |  |  |  |
| milk yield, kg/d | 41.4 | 44.8 | 44.8 | 2.2 | 0.07 |  |
| 3.5% FCM*^e^* | 42.8 | 47.2 | 46.6 | 1.9 | ns |  |
| ECM*^f^* | 42.2 | 46.4 | 46.0 | 1.9 | ns |  |
| milk components |  |  |  |  |  |  |
| fat, kg/d | 1.5 | 1.7 | 1.7 | 0.1 | ns |  |
| fat, % | 3.7 | 3.9 | 3.8 | 0.2 | ns |  |
| protein, kg/d | 1.3 | 1.4 | 1.4 | 0.1 | 0.06 |  |
| protein, % | 3.1 | 3.1 | 3.2 | 0.1 | ns |  |
| lactose, kg/d | 2.3 | 2.5 | 2.5 | 0.1 | 0.07 |  |
| lactose, % | 5.6 | 5.6 | 5.6 | 0.1 | ns |  |
|  |  |  |  |  |  |  |
| fecal output (DM, kg/d) | 7.4 | 7.8 | 8.6 | 0.6 | 0.06 |  |
| feed efficiency*^g^* | 1.5 | 1.7 | 1.7 | 0.2 | ns |  |

*^a^*LS means are based on 6 dairy cows per treatment. *^b^*CON: control (0% of DM as encapsulated echium oil), *^c^*LEO: 1.5% of DM as encapsulated echium oil, and 1.5% of DM as encapsulation matrix, *^d^*HEO: 3% of DM as encapsulated echium oil. *^e^*3.5% FCM = [0.43 x milk yield (kg/d)] + [16.22 x fat yield (kg/d)]. *^f^*ECM = [12.82 x fat yield (kg/d)] + [7.13 x protein yield (kg/d)] + [0.32 x milk yield (kg/d). *^g^*Feed efficiency = kg of 3.5% FCM/kg DMI. ns = non-significant.
