# Supplementary material for: Lipid Encapsulation Provides Insufficient Total-Tract Digestibility to Achieve an Optimal Transfer Efficiency of Fatty Acids to Milk Fat
Source: PLoS One. 2016 Oct 14;11(10):e0164700. doi: 10.1371/journal.pone.0164700 (PMC5065208; doi:10.1371/journal.pone.0164700)
Supplement: S2 Table — (DOCX) [file pone.0164700.s003.docx]

**Supplemental Table 2.** Fatty acid composition*^a^* (g/100g FA) of milk fat by day in response to CON*^b^*, LEO*^c^*, and HEO*^d^* diets.

| **fatty acid** | **Treatment** | | | | | | | | | | | | | | | | | | **SE** | ***P* value** |
| --- | --- | --- | --- | --- | --- | --- | --- | --- | --- | --- | --- | --- | --- | --- | --- | --- | --- | --- | --- | --- |
|  | **CON** | | | **LEO** | | | | | | | | **HEO** | | | | | | |  |  |
|  | **D-**2 | **D-**1 | **D**1 | | **D**2  0**.**17  0**.**12  0**.**30  1**.**59  0**.**75**b**  1**.**33**a**  1**.**39**b**  0**.**70  0**.**00  23**.**1**b**  0**.**63**b**  0**.**09  0**.**40**ab**  0**.**81  2**.**64 | **D**3  39**.**0  0**.**55  0**.**32  0**.**64  1**.**83**ab**  0**.**55  2**.**10  5**.**82  0**.**91  0**.**70  0**.**12  0**.**81  0**.**30  0**.**22  0**.**15 | **D**4  5**.**05**ab**  0**.**36**b**  0**.**02  0**.**19**a**  0**.**06  1**.**39**b**  0**.**09  0**.**18  0**.**08  0**.**00**a**  0**.**12**b**  0**.**18  0**.**41**ab**  0**.**23 | **D**5 | **D**6 | **D**7  0**.**17**a**  0**.**26  0**.**05**b**  0**.**10  1**.**29  67**.**6**ab**  17**.**82  5**.**98  8**.**60  5**.**87**ab**  1**.**87**a**  3**.**12**b**  0**.**35  4**.**51**a** | **D**8 | | **D**9 | **D**10  39**.**1  0**.**47  0**.**30  0**.**58  2**.**02**b**  0**.**55  1**.**99  5**.**33  0**.**85  0**.**70  0**.**11  0**.**88  0**.**33  0**.**23  0**.**19 | **D**11  4**.**30**b**  0**.**42**ab**  0**.**02  0**.**29**b**  0**.**09  1**.**56**c**  0**.**10  0**.**14  0**.**07  0**.**03**b**  0**.**23**c**  0**.**21  0**.**36**b**  0**.**16 | **D**12 | **D**13 | **D**14  0**.**25**b**  0**.**31  0**.**04**b**  0**.**17  1**.**33  69**.**4**b**  16**.**83  5**.**88  8**.**54  5**.**10**b**  2**.**28**b**  2**.**26**c**  0**.**37  3**.**77**b** |  |  |
| 4:0 | 3.12 | 3.13 | 3.10 | | 3.01 | 3.09 | 3.08 | 3.11 | 3.12 | 3.13 | 3.33 | | 3.06 | 3.06 | 3.00 | 3.08 | 3.07 | 3.27 | 0.10 | <0.01 |
| 5:0 | 0.02 | 0.01 | 0.02 | | 0.02 | 0.01 | 0.02 | 0.02 | 0.02 | 0.01 | 0.02 | | 0.02 | 0.01 | 0.02 | 0.02 | 0.02 | 0.01 | 0.00 | ns |
| 6:0 | 2.00 | 2.06 | 2.04 | | 1.98 | 1.99 | 1.99 | 2.01 | 2.04 | 2.04 | 2.06 | | 1.98 | 2.01 | 1.95 | 1.98 | 1.98 | 2.09 | 0.06 | <0.01 |
| 7:0 | 0.01 | 0.01 | 0.02 | | 0.02 | 0.02 | 0.02 | 0.02 | 0.02 | 0.02 | 0.01 | | 0.02 | 0.02 | 0.02 | 0.02 | 0.03 | 0.02 | 0.00 | 0.057 |
| 8:0 | 1.20 | 1.24 | 1.23 | | 1.18 | 1.20 | 1.21 | 1.21 | 1.24 | 1.25 | 1.19 | | 1.21 | 1.24 | 1.20 | 1.21 | 1.19 | 1.26 | 0.04 | <0.01 |
| 9:0 | 0.02 | 0.02 | 0.03 | | 0.03 | 0.03 | 0.03 | 0.03 | 0.03 | 0.03 | 0.03 | | 0.03 | 0.03 | 0.03 | 0.03 | 0.03 | 0.03 | 0.00 | ns |
| 10:0 | 2.83 | 2.93 | 2.97 | | 2.82 | 2.79 | 2.85 | 2.83 | 2.89 | 2.96 | 2.78 | | 2.89 | 2.95 | 2.87 | 2.88 | 2.86 | 2.93 | 0.09 | <0.01 |
| 11:0 | 0.26 | 0.28 | 0.29 | | 0.29 | 0.28 | 0.28 | 0.30 | 0.30 | 0.28 | 0.32 | | 0.28 | 0.26 | 0.32 | .033 | 0.31 | 0.28 | 0.01 | <0.001 |
| 12:0 | 3.35 | 3.46 | 3.51 | | 3.38 | 3.32 | 3.38 | 3.36 | 3.43  443 | 3.50 | 3.33 | | 3.44 | 3.51 | 3.43 | 3.42 | 3.37 | 3.42 | 0.11 | ns |
| *iso* 13:0 | 0.03 | 0.03 | 0.02 | | 0.03 | 0.03 | 0.03 | 0.03 | 0.03 | 0.03 | 0.02 | | 0.02 | 0.03 | 0.03 | 0.03 | 0.03 | 0.03 | 0.00 | ns |
| *anteiso* 13:0 | 0.08 | 0.09 | 0.09 | | 0.09 | 0.08 | 0.08 | 0.09 | 0.09 | 0.09 | 0.08 | | 0.08 | 0.09 | 0.09 | 0.09 | 0.08 | 0.08 | 0.01 | ns |
| 13:0 | 0.17 | 0.18 | 0.19 | | 0.19 | 0.18 | 0.18 | 0.18 | 0.19 | 0.18 | 0.18 | | 0.19 | 0.19 | 0.19 | 0.18 | 0.18 | 0.18 | 0.01 | ns |
| *iso* 14:0 | 0.11 | 0.10 | 0.10 | | 0.09 | 0.08 | 0.09 | 0.08 | 0.08 | 0.09 | 0.07 | | 0.07 | 0.07 | 0.07 | 0.08 | 0.09 | 0.09 | 0.01 | <0.05 |
| 14:0 | 12.5 | 12.5 | 12.4 | | 12.1 | 12.1 | 12.1 | 12.2 | 12.1 | 12.4 | 12.1 | | 12.2 | 12.0 | 12.0 | 12.0 | 11.9 | 12.0 | 0.31 | <0.05 |
| *iso* 15:0 | 0.20 | 0.18 | 0.18 | | 0.18 | 0.18 | 0.18 | 0.17 | 0.17 | 0.17 | 0.16 | | 0.17 | 0.17 | 0.17 | 0.18 | 0.19 | 0.18 | 0.01 | <0.001 |
| 14:1 *t*9 | 0.01 | 0.01 | 0.01 | | 0.01 | 0.01 | 0.01 | 0.01 | 0.02 | 0.01 | 0.01 | | 0.01 | 0.01 | 0.01 | 0.01 | 0.01 | 0.01 | 0.01 | ns |
| *anteiso* 15:0 | 0.47 | 0.46 | 0.45 | | 0.44 | 0.44 | 0.43 | 0.42 | 0.42 | 0.41 | 0.41 | | 0.42 | 0.42 | 0.43 | 0.43 | 0.43 | 0.42 | 0.02 | 0.053 |
| 14:1 *c*9 | 0.93 | 0.96 | 1.02 | | 1.03 | 1.02 | 1.01 | 1.05 | 1.05 | 1.00 | 0.97 | | 0.97 | 0.91 | 0.91 | 0.93 | 0.90 | 0.91 | 0.07 | ns |
| 15:0 | 0.95 | 0.96 | 1.01 | | 1.00 | 0.97 | 0.96 | 0.94 | 0.92 | 0.91 | 0.93 | | 0.96 | 0.96 | 0.95 | 0.96 | 0.96 | 0.94 | 0.01 | ns |
| *iso* 16:0 | 0.25 | 0.25 | 0.23 | | 0.22 | 0.19 | 0.19 | 0.20 | 0.21 | 0.21 | 0.21 | | 0.18 | 0.19 | 0.20 | 0.21 | 0.23 | 0.21 | 0.01 | <0.01 |
| 16:0 | 32.5 | 32.8 | 33.6 | | 33.4 | 33.5 | 33.7 | 33.7 | 32.6 | 32.7 | 32.8 | | 32.5 | 31.8 | 31.6 | 31.2 | 31.4 | 31.6 | 0.89 | <0.01 |
| *iso* 17:0 | 0.33 | 0.34 | 0.32 | | 0.32 | 0.33 | 0.32 | 0.32 | 0.32 | 0.31 | 0.31 | | 0.31 | 0.32 | 0.32 | 0.31 | 0.32 | 0.31 | 0.02 | ns |
| 16:1 *t*9 | 0.02 | 0.02 | 0.01 | | 0.02 | 0.01 | 0.01 | 0.02 | 0.01 | 0.01 | 0.02 | | 0.02 | 0.02 | 0.02 | 0.02 | 0.02 | 0.02 | 0.00 | ns |
| 16:1 isomer | 0.00 | 0.01 | 0.00 | | 0.00 | 0.01 | 0.00 | 0.01 | 0.00 | 0.01 | 0.01 | | 0.00 | 0.01 | 0.01 | 0.01 | 0.01 | 0.01 | 0.00 | <0.05 |
| 16:1 *c*7 | 0.03 | 0.03 | 0.03 | | 0.03 | 0.03 | 0.03 | 0.03 | 0.03 | 0.03 | 0.03 | | 0.03 | 0.03 | 0.03 | 0.03 | 0.03 | 0.03 | 0.00 | ns |
| 16:1 *c*8 | 0.14 | 0.14 | 0.14 | | 0.14 | 0.14 | 0.13 | 0.13 | 0.13 | 0.12 | 0.12 | | 0.12 | 0.12 | 0.12 | 0.12 | 0.12 | 0.12 | 0.01 | ns |
| *anteiso* 17:0 | 0.02 | 0.02 | 0.01 | | 0.01 | 0.02 | 0.02 | 0.02 | 0.02 | 0.02 | 0.02 | | 0.02 | 0.02 | 0.02 | 0.02 | 0.01 | 0.02 | 0.00 | ns |
| 16:1 *c*9 | 1.78 | 1.80 | 1.88 | | 1.92 | 1.89 | 1.85 | 1.88 | 1.85 | 1.76 | 1.75 | | 1.74 | 1.65 | 1.64 | 1.63 | 1.61 | 1.60 | 0.11 | <0.05 |
| 16:1 *c*10/*t*13 | 0.01 | 0.01 | 0.01 | | 0.01 | 0.02 | 0.01 | 0.01 | 0.01 | 0.01 | 0.03 | | 0.02 | 0.03 | 0.03 | 0.03 | 0.03 | 0.01 | 0.00 | <0.001 |
| 16:1 *c*11 | 0.09 | 0.08 | 0.07 | | 0.06 | 0.07 | 0.06 | 0.06 | 0.06 | 0.07 | 0.02 | | 0.06 | 0.06 | 0.03 | 0.02 | 0.02 | 0.06 | 0.00 | <0.001 |
| 17:0 | 0.62 | 0.61 | 0.61 | | 0.60 | 0.59 | 0.57 | 0.57 | 0.57 | 0.58 | 0.57 | | 0.57 | 0.57 | 0.57 | 0.58 | 0.58 | 0.57 | 0.01 | ns |
| *iso* 18:0 | 0.01 | 0.01 | 0.01 | | 0.02 | 0.01 | 0.01 | 0.01 | 0.01 | 0.01 | 0.01 | | 0.01 | 0.01 | 0.01 | 0.01 | 0.01 | 0.01 | 0.00 | ns |
| 17:1 *c*8 | 0.18 | 0.17 | 0.17 | | 0.17 | 0.16 | 0.16 | 0.16 | 0.15 | 0.15 | 0.14 | | 0.15 | 0.14 | 0.13 | 0.14 | 0.15 | 0.13 | 0.01 | 0.075 |
| 18:0 | 9.70 | 9.34 | 8.61 | | 8.84 | 8.93 | 8.97 | 8.70 | 8.97 | 9.4 | 9.07 | | 9.28 | 9.59 | 9.75 | 9.89 | 10.0 | 9.76 | 0.34 | ns |
| 18:1 *t*4 | 0.01 | 0.01 | 0.01 | | 0.01 | 0.01 | 0.01 | 0.01 | 0.01 | 0.01 | 0.01 | | 0.01 | 0.01 | 0.01 | 0.01 | 0.02 | 0.01 | 0.00 | ns |
| 18:1 *t*5 | 0.01 | 0.01 | 0.02 | | 0.01 | 0.01 | 0.01 | 0.01 | 0.01 | 0.01 | 0.01 | | 0.01 | 0.01 | 0.02 | 0.02 | 0.02 | 0.01 | 0.00 | <0.05 |
| 18:1 *t*6-8 | 0.24 | 0.26 | 0.26 | | 0.25 | 0.26 | 0.25 | 0.26 | 0.26 | 0.27 | 0.28 | | 0.28 | 0.28 | 0.28 | 0.28 | 0.25 | 0.27 | 0.01 | <0.01 |
| 18:1 *t*9 | 0.21 | 0.19 | 0.22 | | 0.22 | 0.21 | 0.21 | 0.22 | 0.22 | 0.23 | 0.24 | | 0.23 | 0.24 | 0.25 | 0.25 | 0.24 | 0.23 | 0.01 | ns |
| 18:1 *t*10 | 0.29 | 0.29 | 0.31 | | 0.31 | 0.32 | 0.30 | 0.33 | 0.33 | 0.34 | 0.39 | | 0.36 | 0.38 | 0.42 | 0.37 | 0.35 | 0.36 | 0.02 | <0.01 |
| 18:1 *t*11 | 0.98 | 0.92 | 0.89 | | 0.93 | 0.99 | 0.98 | 1.00 | 1.09 | 1.14 | 1.15 | | 1.17 | 1.26 | 1.25 | 1.24 | 1.21 | 1.19 | 0.09 | <0.001 |
| 18:1 *t*12 | 0.31 | 0.32 | 0.34 | | 0.35 | 0.36 | 0.34 | 0.36 | 0.38 | 0.39 | 0.41 | | 0.41 | 0.44 | 0.44 | 0.42 | 0.38 | 0.40 | 0.02 | <0.001 |
| 18:1 *t*13/14/*c*6-8 | 0.40 | 0.42 | 0.44 | | 0.47 | 0.48 | 0.46 | 0.49 | 0.50 | 0.52 | 0.63 | | 0.54 | 0.62 | 0.66 | 0.63 | 0.60 | 0.54 | 0.03 | <0.001 |
| 18:1 *c*9 | 17.7 | 1.76 | 17.3 | | 17.9 | 17.7 | 17.5 | 17.4 | 17.8 | 17.3 | 17.5 | | 17.5 | 17.5 | 17.5 | 17.7 | 17.7 | 17.4 | 0.69 | 0.067 |
| 18:1 *c*11 | 0.57 | 0.57 | 0.55 | | 0.54 | 0.51 | 0.49 | 0.50 | 0.48 | 0.49 | 0.52 | | 0.50 | 0.49 | 0.51 | 0.51 | 0.51 | 0.46 | 0.03 | <0.05 |
| 18:1 *c*12 | 0.25 | 0.26 | 0.28 | | 0.28 | 0.29 | 0.27 | 0.28 | 0.28 | 0.30 | 0.33 | | 0.31 | 0.32 | 0.33 | 0.33 | 0.30 | 0.30 | 0.02 | 0.062 |
| 18:1 *c*13 | 0.05 | 0.05 | 0.05 | | 0.05 | 0.05 | 0.05 | 0.04 | 0.05 | 0.04 | 0.04 | | 0.04 | 0.04 | 0.04 | 0.04 | 0.04 | 0.03 | 0.01 | ns |
| 18:1 *c*14/*t*16 | 0.26 | 0.26 | 0.27 | | 0.29 | 0.29 | 0.28 | 0.28 | 0.30 | 0.29 | 0.30 | | 0.32 | 0.34 | 0.35 | 0.34 | 0.33 | 0.34 | 0.01 | <0.001 |
| 18:1 *c*15 | 0.05 | 0.05 | 0.06 | | 0.07 | 0.07 | 0.07 | 0.07 | 0.07 | 0.07 | 0.09 | | 0.08 | 0.11 | 0.11 | 0.11 | 0.11 | 0.09 | 0.01 | ns |
| 18:2 *t*10*,t*14 | 0.08 | 0.08 | 0.07 | | 0.08 | 0.08 | 0.06 | 0.07 | 0.07 | 0.07 | 0.07 | | 0.07 | 0.07 | 0.06 | 0.07 | 0.07 | 0.08 | 0.01 | ns |
| 18:2 *t*9*,t*12 | 0.16 | 0.16 | 0.18 | | 0.20 | 0.20 | 0.20 | 0.21 | 0.21 | 0.22 | 0.23 | | 0.23 | 0.24 | 0.24 | 0.24 | 0.22 | 0.22 | 0.01 | ns |
| cyclohexyl-11 11:0 | 0.14 | 0.14 | 0.14 | | 0.14 | 0.13 | 0.14 | 0.14 | 0.13 | 0.14 | 0.17 | | 0.15 | 0.14 | 0.15 | 0.16 | 0.16 | 0.12 | 0.01 | <0.05 |
| 18:2 *c*9*,t*14 | 0.10 | 0.10 | 0.11 | | 0.13 | 0.11 | 0.12 | 0.11 | 0.12 | 0.11 | 0.12 | | 0.12 | 0.13 | 0.12 | 0.13 | 0.11 | 0.11 | 0.01 | ns |
| 18:1 *c*16 | 0.05 | 0.04 | 0.05 | | 0.05 | 0.05 | 0.05 | 0.06 | 0.06 | 0.05 | 0.06 | | 0.07 | 0.06 | 0.08 | 0.07 | 0.07 | 0.06 | 0.00 | ns |
| 18:2 *t*9*,c*12 | 0.07 | 0.05 | 0.06 | | 0.09 | 0.12 | 0.13 | 0.13 | 0.15 | 0.15 | 0.14 | | 0.19 | 0.24 | 0.26 | 0.24 | 0.23 | 0.23 | 0.01 | <0.001 |
| 18:2 *t*11*,c*15 | 0.02 | 0.02 | 0.02 | | 0.02 | 0.02 | 0.02 | 0.02 | 0.02 | 0.02 | 0.02 | | 0.02 | 0.02 | 0.02 | 0.03 | 0.02 | 0.02 | 0.00 | ns |
| 18:2 *c*9*,c*12 | 1.99 | 1.94 | 1.94 | | 1.89 | 1.88 | 1.88 | 1.95 | 2.03 | 2.02 | 2.11 | | 2.02 | 2.05 | 2.06 | 2.09 | 2.05 | 2.06 | 0.09 | <0.001 |
| 18:2 *t*12*,c*15 | 0.07 | 0.07 | 0.06 | | 0.06 | 0.05 | 0.06 | 0.06 | 0.07 | 0.06 | 0.06 | | 0.06 | 0.06 | 0.05 | 0.06 | 0.05 | 0.06 | 0.01 | ns |
| 20 :0 | 0.13 | 0.12 | 0.11 | | 0.12 | 0.12 | 0.12 | 0.11 | 0.11 | 0.12 | 0.10 | | 0.12 | 0.12 | 0.11 | 0.11 | 0.11 | 0.12 | 0.01 | 0.062 |
| 18:3 *c*6*,c*9*,c*12 | 0.02 | 0.02 | 0.02 | | 0.03 | 0.04 | 0.04 | 0.04 | 0.05 | 0.04 | 0.04 | | 0.06 | 0.06 | 0.06 | 0.07 | 0.07 | 0.07 | 0.01 | <0.01 |
| 20:1 *c*9 | 0.07 | 0.08 | 0.07 | | 0.08 | 0.02 | 0.02 | 0.03 | 0.02 | 0.05 | 0.09 | | 0.03 | 0.02 | 0.09 | 0.09 | 0.10 | 0.05 | 0.01 | <0.001 |
| 18:3 *c*9*,c*12*,c*15 | 0.33 | 0.33 | 0.35 | | 0.38 | 0.46  46 | 0.47 | 0.47 | 0.49 | 0.47 | 0.49 | | 0.53 | 0.56 | 0.58 | 0.58 | 0.62 | 0.63 | 0.02 | <0.001 |
| 18:2 *c*9*,t*11 | 0.45 | 0.44 | 0.46 | | 0.49 | 0.50 | 0.49 | 0.52 | 0.55 | 0.55 | 0.56 | | 0.57 | 0.59 | 0.61 | 0.58 | 0.56 | 0.56 | 0.04 | <0.05 |
| 18:2 *c*9*,c*11 | 0.02 | 0.02 | 0.02 | | 0.02 | 0.02 | 0.02 | 0.02 | 0.02 | 0.02 | 0.02 | | 0.02 | 0.02 | 0.02 | 0.02 | 0.02 | 0.02 | 0.00 | ns |
| 18:2 *t*7*,t*9/*t*10*,t*12 | 0.01 | 0.01 | 0.02 | | 0.01 | 0.01 | 0.01 | 0.01 | 0.02 | 0.01 | 0.01 | | 0.02 | 0.02 | 0.02 | 0.02 | 0.02 | 0.02 | 0.00 | ns |
| 18:4 *c*6*,c*9*,c*12*,c*15 | 0.00 | 0.00 | 0.03 | | 0.04 | 0.04 | 0.04 | 0.05 | 0.05 | 0.05 | 0.06 | | 0.06 | 0.08 | 0.08 | 0.07 | 0.09 | 0.09 | 0.00 | <0.001 |
| 20:2 *c*11*,c*14 | 0.02 | 0.02 | 0.02 | | 0.02 | 0.02 | 0.02 | 0.02 | 0.02 | 0.02 | 0.03 | | 0.02 | 0.02 | 0.04 | 0.04 | 0.03 | 0.02 | 0.00 | <0.01 |
| 20:3 *c*8*,c*11*,c*14 | 0.16 | 0.15 | 0.15 | | 0.14  .14 | 0.14 | 0.13 | 0.14 | 0.15 | 0.14 | 0.11 | | 0.14 | 0.15 | 0.10 | 0.11 | 0.12 | 0.03 | 0.01 | <0.001 |
| 22:1 *c*13 | 0.01 | 0.01 | 0.02 | | 0.02 | 0.02 | 0.02 | 0.02 | 0.02 | 0.02 | 0.01 | | 0.02 | 0.02 | 0.01 | 0.01 | 0.02 | 0.03 | 0.00 | <0.05 |
| 20:3 *c*11*,c*14*,c*17 | 0.00 | 0.01 | 0.01 | | 0.01 | 0.01 | 0.01 | 0.01 | 0.01 | 0.01 | 0.01 | | 0.01 | 0.01 | 0.02 | 0.01 | 0.02 | 0.00 | 0.00 | <0.05 |
| 20:4 *c*5*,c*8*,c*11*,c*14 | 0.14 | 0.13 | 0.13 | | 0.13 | 0.13 | 0.13 | 0.12 | 0.13 | 0.13 | 0.12 | | 0.13 | 0.13 | 0.11 | 0.12 | 0.12 | 0.13 | 0.01 | <0.05 |
| 23:0 | 0.01 | 0.01 | 0.01 | | 0.01 | 0.00 | 0.01 | 0.01 | 0.01 | 0.01 | 0.00  .0. | | 0.02 | 0.02 | 0.00 | 0.02 | 0.02 | 0.02 | 0.00 | <0.01 |
| 20:5 *c*5*,c*8*,c*11*,c*14*,c*17 | 0.03 | 0.03 | 0.03 | | 0.03 | 0.03 | 0.03 | 0.03 | 0.03 | 0.03 | 0.03 | | 0.03 | 0.04 | 0.04 | 0.05 | 0.04 | 0.04 | 0.00 | <0.05 |
| 22:4 *c*7*,c*10*,c*13*,c*16 | 0.02 | 0.03 | 0.02 | | 0.02 | 0.02 | 0.02 | 0.02 | 0.03 | 0.02 | 0.02 | | 0.03 | 0.02 | 0.02 | 0.03 | 0.02 | 0.02 | 0.00 | ns |
| 22:5 *c*7*,c*10*,c*13*,c*16*,c*19 | 0.05 | 0.04 | 0.04 | | 0.04 | 0.04 | 0.04 | 0.05 | 0.04 | 0.04 | 0.04 | | 0.04 | 0.04 | 0.04 | 0.05 | 0.05 | 0.05 | 0.00 | ns |
| unknown | 0.53 | 0.49 | 0.50 | | 0.47 | 0.43 | 0.44 | 0.48 | 0.49 | 0.46 | 0.29 | | 0.44 | 0.51 | 0.59 | 0.61 | 0.62 | 0.84 | 0.02 | <0.001 |
| *de novo^e^* | 27.4 | 27.8 | 27.9 | | 27.0 | 27.0 | 27.1 | 27.2 | 27.4 | 27.7 | 27.2 | | 27.2 | 27.2 | 26.9 | 27.0 | 26.9 | 27.3 | 0.46 | ns |
| mixed*^f^* | 34.8 | 35.1 | 35.9 | | 35.8 | 35.9 | 36.0 | 36.0 | 34.9 | 35.0 | 34.9 | | 34.7 | 33.9 | 33.7 | 33.3 | 33.5 | 33.7 | 0.96 | <0.01 |
| preformed*^g^* | 35.3 | 34.8 | 33.9 | | 34.9 | 34.9 | 34.7 | 34.6 | 35.5 | 35.2 | 35.9 | | 36.0 | 36.7 | 37.1 | 37.4 | 37.3 | 36.4 | 1.21 | <0.01 |
| total SFA*^h^* | 69.4 | 69.7 | 69.7 | | 69.0 | 69.2 | 69.5 | 69.3 | 68.6 | 69.1 | 68.8 | | 68.8 | 68.4 | 68.1 | 68.0 | 68.2 | 68.5 | 0.97 | <0.05 |
| total MUFA*^i^* | 24.7 | 24.6 | 24.5 | | 25.2 | 25.0 | 24.7 | 24.8 | 25.2 | 24.7 | 25.2 | | 25.0 | 25.1 | 25.3 | 25.4 | 25.1 | 24.8 | 0.80 | 0.056 |
| total *trans* 18:1 | 2.05 | 2.00 | 2.05 | | 2.08 | 2.16 | 2.10 | 2.20 | 2.31 | 2.40 | 2.49 | | 2.47 | 2.63 | 2.66 | 2.58 | 2.46 | 2.48 | 0.16 | <0.001 |
| total PUFA*^j^* | 3.72 | 3.65 | 3.73 | | 3.82 | 3.93 | 3.89 | 4.05 | 4.26 | 4.19 | 4.25 | | 4.38 | 4.55 | 4.52 | 4.58 | 4.52 | 4.44 | 0.18 | <0.001 |
| total n-6 PUFA*^k^* | 2.32 | 2.27 | 2.27 | | 2.21 | 2.22 | 2.19 | 2.28 | 2.39 | 2.36 | 2.39 | | 2.38 | 2.41 | 2.35 | 2.41 | 2.38 | 2.31 | 0.10 | <0.001 |
| total n-3 PUFA*^l^* | 0.41 | 0.41 | 0.44 | | 0.50 | 0.57 | 0.58 | 0.60 | 0.62 | 0.60 | 0.62 | | 0.68 | 0.72 | 0.74 | 0.76 | 0.81 | 0.81 | 0.03 | <0.001 |
| total CLA*^m^* | 0.48 | 0.47 | 0.49 | | 0.52 | 0.53 | 0.52 | 0.55 | 0.58 | 0.59 | 0.57 | | 0.61 | 0.63 | 0.63 | 0.60 | 0.59 | 0.59 | 0.04 | <0.05 |

*^a^*Means are based on 6 dairy cattle per treatment. *^b^*CON: control (0% of DM as lipid-encapsulated echium oil), *^c^*LEO: 1.5% of DM as lipid-encapsulated echium oil, 1.5% of DM as encapsulation, *^d^*HEO: 3% of DM as lipid-encapsulated echium oil. *^e^De novo*: sum of fatty acids < 16 carbons. *^f^*Mixed: sum of 16:0 and 16:1 c9. *^g^*Preformed: sum of fatty acids >16 carbons. *^h^*Total SFA: sum of saturated fatty acids (4:0 to 26:0). *^i^*Total MUFA: sum of monounsaturated fatty acids (14:1 to 24:1). *^j^*Total PUFA: sum of polyunsaturated fatty acids (18:2 to 22:5). *^k^*Total n-6 PUFA: sum of n-6 PUFA: 18:2 *c*9*,c*12, 18:3 *c*6*,c*9*,c*12, 20:2 *c*11*,c*14, 20:3 *c*8*,c*11*,c*14, 20:4 *c*5*,c*8*,c*11*,c*14and 22:4 *c*7*,c*10*,c*13*,c*16. *^l^*Total n-3 PUFA: sum of n-3 PUFA: 18:3 *c*9*,c*12*,c*15, 18:4 *c*6*,c*9*,c*12*,c*15, 20:5 *c*5*,c*8*,c*11*,c*14*,c*17, and 22:5 *c*7*,c*10*,c*13*,c*16*,c*19. *^m^*Total CLA: sum of conjugated linoleic acids: 18:2 *c*9*,t*11, 18:2 *c*9*,c*11, 18:2 *t*7*,t*9, and 18:2 *t*10*,t*12.
