# Supplementary material for: Lipid Encapsulation Provides Insufficient Total-Tract Digestibility to Achieve an Optimal Transfer Efficiency of Fatty Acids to Milk Fat
Source: PLoS One. 2016 Oct 14;11(10):e0164700. doi: 10.1371/journal.pone.0164700 (PMC5065208; doi:10.1371/journal.pone.0164700)
Supplement: S3 Table — (DOCX) [file pone.0164700.s004.docx]

**Supplemental Table 3.** Fatty acid composition*^a^* (g/100g FA) of the cholesterol ester plasma lipid fraction by day in response to CON*^b^*, LEO*^c^*, and HEO*^d^* diets.

| **fatty acid** | **Treatment** | | | | | | | | | | | **SE** | ***P* value** |
| --- | --- | --- | --- | --- | --- | --- | --- | --- | --- | --- | --- | --- | --- |
|  | **CON** | | **LEO** | | | | | **HEO** | | | |  |  |
|  | **D-**1 | **D**2  0**.**17  0**.**12  0**.**30  1**.**59  0**.**75**b**  1**.**33**a**  1**.**39**b**  0**.**70  0**.**00  23**.**1**b**  0**.**63**b**  0**.**09  0**.**40**ab**  0**.**81  2**.**64 | | **D**3  39**.**0  0**.**55  0**.**32  0**.**64  1**.**83**ab**  0**.**55  2**.**10  5**.**82  0**.**91  0**.**70  0**.**12  0**.**81  0**.**30  0**.**22  0**.**15 | **D**4  5**.**05**ab**  0**.**36**b**  0**.**02  0**.**19**a**  0**.**06  1**.**39**b**  0**.**09  0**.**18  0**.**08  0**.**00**a**  0**.**12**b**  0**.**18  0**.**41**ab**  0**.**23 | **D**7  0**.**17**a**  0**.**26  0**.**05**b**  0**.**10  1**.**29  67**.**6**ab**  17**.**82  5**.**98  8**.**60  5**.**87**ab**  1**.**87**a**  3**.**12**b**  0**.**35  4**.**51**a** | **D**9  0**.**18  0**.**06  0**.**24  1**.**47  0**.**63**c**  1**.**06**b**  1**.**23**c**  0**.**60  0**.**00  25**.**3**c**  0**.**54**b**  0**.**08  0**.**30**b**  0**.**70  2**.**24 | | **D**10  39**.**1  0**.**47  0**.**30  0**.**58  2**.**02**b**  0**.**55  1**.**99  5**.**33  0**.**85  0**.**70  0**.**11  0**.**88  0**.**33  0**.**23  0**.**19 | **D**11  4**.**30**b**  0**.**42**ab**  0**.**02  0**.**29**b**  0**.**09  1**.**56**c**  0**.**10  0**.**14  0**.**07  0**.**03**b**  0**.**23**c**  0**.**21  0**.**36**b**  0**.**16 | **D**14  0**.**25**b**  0**.**31  0**.**04**b**  0**.**17  1**.**33  69**.**4**b**  16**.**83  5**.**88  8**.**54  5**.**10**b**  2**.**28**b**  2**.**26**c**  0**.**37  3**.**77**b** |  |  |
| 10:0 | 0.00 | 0.79 | | 0.98 | 1.31 | 1.10 | 1.07 | | 1.80 | 0.60 | 0.63 | 0.33 | <0.05 |
| 12:0 | 0.07 | 0.05 | | 0.06 | 0.06 | 0.06 | 0.05 | | 0.04 | 0.05 | 0.07 | 0.01 | ns |
| *iso* 13:0 | 0.00 | 0.01 | | 0.00 | 0.01 | 0.00 | 0.00 | | 0.01 | 0.01 | 0.00 | 0.00 | ns |
| 13:0 | 0.03 | 0.03 | | 0.02 | 0.02 | 0.03 | 0.02 | | 0.01 | 0.01 | 0.04 | 0.01 | ns |
| *iso* 14:0 | 0.11 | 0.08 | | 0.14 | 0.17 | 0.09 | 0.14 | | 0.60 | 0.10 | 0.08 | 0.15 | ns |
| 14:0 | 1.03 | 0.97 | | 0.91 | 0.90 | 0.90 | 0.84 | | 0.81 | 0.88 | 0.88 | 0.06 | <0.01 |
| *iso* 15:0 | 0.49 | 0.49 | | 0.47 | 0.47 | 0.45 | 0.43 | | 0.41 | 0.45 | 0.42 | 0.03 | <0.01 |
| *anteiso* 15:0 | 0.93 | 0.87 | | 0.86 | 0.84 | 0.82 | 0.77 | | 0.75 | 0.78 | 0.74 | 0.05 | <0.001 |
| 14:1 *c*9 | 0.02 | 0.02 | | 0.02 | 0.00 | 0.01 | 0.01 | | 0.02 | 0.01 | 0.01 | 0.01 | ns |
| 15:0 | 0.64 | 0.58 | | 0.56 | 0.57 | 0.54 | 0.50 | | 0.55 | 0.51 | 0.46 | 0.03 | <0.01 |
| *iso* 16:0 | 0.30 | 0.27 | | 0.25 | 0.24 | 0.24 | 0.23 | | 0.23 | 0.24 | 0.20 | 0.01 | <0.001 |
| 16:0 | 4.23 | 3.48 | | 3.36 | 3.40 | 3.20 | 3.43 | | 3.28 | 3.41 | 3.35 | 0.12 | <0.001 |
| *iso* 17:0 | 0.10 | 0.09 | | 0.09 | 0.10 | 0.12 | 0.10 | | 0.09 | 0.09 | 0.08 | 0.01 | ns |
| 16:1 *t*9 | 0.00 | 0.00 | | 0.01 | 0.02 | 0.02 | 0.02 | | 0.01 | 0.01 | 0.02 | 0.00 | <0.05 |
| 16:1 *c*7 | 0.02 | 0.04 | | 0.03 | 0.04 | 0.03 | 0.04 | | 0.03 | 0.02 | 0.05 | 0.01 | ns |
| 16:1 *c*8 | 0.58 | 0.57 | | 0.58 | 0.58 | 0.59 | 0.51 | | 0.49 | 0.52 | 0.49 | 0.04 | 0.062 |
| *anteiso* 17:0 | 0.01 | 0.03 | | 0.02 | 0.03 | 0.04 | 0.03 | | 0.02 | 0.02 | 0.04 | 0.02 | ns |
| 16:1 *c*9 | 1.26 | 1.23 | | 1.21 | 1.21 | 1.13 | 1.09 | | 1.02 | 1.10 | 1.03 | 0.05 | <0.001 |
| 16:1 *c*10/*t*13 | 0.02 | 0.04 | | 0.02 | 0.03 | 0.02 | 0.03 | | 0.03 | 0.04 | 0.03 | 0.01 | ns |
| 16:1 *c*11 | 0.08 | 0.09 | | 0.06 | 0.05 | 0.07 | 0.06 | | 0.07 | 0.06 | 0.04 | 0.01 | ns |
| 17:0 | 0.09 | 0.05 | | 0.05 | 0.04 | 0.08 | 0.05 | | 0.06 | 0.05 | 0.09 | 0.01 | 0.068 |
| *iso* 18:0 | 0.76 | 0.74 | | 0.72 | 0.70 | 0.64 | 0.63 | | 0.65 | 0.63 | 0.53 | 0.08 | <0.001 |
| 17:1 *c*8 | 0.21 | 0.18 | | 0.22 | 0.18 | 0.21 | 0.18 | | 0.18 | 0.17 | 0.14 | 0.01 | <0.05 |
| 18:0 | 2.32 | 0.74 | | 0.51 | 0.50 | 2.10 | 0.72 | | 0.61 | 0.62 | 0.58 | 0.11 | <0.001 |
| 18:1 *t*5 | 0.00 | 0.01 | | 0.00 | 0.01 | 0.00 | 0.02 | | 0.00 | 0.00 | 0.00 | 0.01 | ns |
| 18:1 *t*6-8 | 0.03 | 0.05 | | 0.01 | 0.03 | 0.06 | 0.04 | | 0.04 | 0.01 | 0.04 | 0.01 | ns |
| 18:1 *t*9 | 0.03 | 0.05 | | 0.01 | 0.04 | 0.03 | 0.03 | | 0.02 | 0.02 | 0.04 | 0.01 | ns |
| 18:1 *t*10 | 0.07 | 0.04 | | 0.02 | 0.07 | 0.06 | 0.04 | | 0.03 | 0.03 | 0.04 | 0.01 | <0.05 |
| 18:1 *t*11 | 0.10 | 0.05 | | 0.01 | 0.04 | 0.13 | 0.06 | | 0.03 | 0.03 | 0.03 | 0.01 | <0.01 |
| 18:1 *t*12 | 0.10 | 0.12 | | 0.05 | 0.05 | 0.10 | 0.08 | | 0.08 | 0.06 | 0.08 | 0.01 | ns |
| 18:1 *t*13/14/*c*6-8 | 0.19 | 0.16 | | 0.13 | 0.15 | 0.22 | 0.17 | | 0.15 | 0.11 | 0.16 | 0.02 | 0.069 |
| 18:1 *c*9 | 2.80 | 2.68 | | 2.57 | 2.60 | 2.65 | 2.50 | | 2.32 | 2.45 | 2.37 | 0.08 | <0.001 |
| 18:1 *c*11 | 0.28 | 0.25 | | 0.22 | 0.22 | 0.20 | 0.22 | | 0.17 | 0.20 | 0.19 | 0.02 | <0.01 |
| 18:1 *c*12 | 0.20 | 0.21 | | 0.20 | 0.21 | 0.18 | 0.22 | | 0.18 | 0.20 | 0.20 | 0.02 | ns |
| 18:2 *t*10*,t*14 | 0.07 | 0.04 | | 0.06 | 0.07 | 0.07 | 0.07 | | 0.07 | 0.05 | 0.08 | 0.02 | ns |
| 18:2 *t*9*,t*12 | 0.07 | 0.10 | | 0.08 | 0.02 | 0.04 | 0.47 | | 0.24 | 0.53 | 0.26 | 0.07 | <0.01 |
| cyclohexyl-11 11:0 | 0.55 | 0.58 | | 0.55 | 0.51 | 0.53 | 0.51 | | 0.50 | 0.53 | 0.44 | 0.02 | <0.05 |
| 18:2 *c*12*,t*16 | 0.00 | 0.00 | | 0.02 | 0.03 | 0.02 | 0.04 | | 0.06 | 0.04 | 0.03 | 0.01 | <0.01 |
| 18:2 *c*9*,c*12 | 71.19 | 72.02 | | 72.39 | 71.69 | 69.09 | 71.38 | | 71.10 | 71.83 | 71.14 | 0.80 | ns |
| 18:3 *c*6*,c*9*,c*12 | 1.71 | 1.93 | | 1.99 | 2.04 | 2.06 | 2.17 | | 2.19 | 2.34 | 2.48 | 0.10 | <0.001 |
| 18:3 *c*9*,c*12*,c*15 | 6.67 | 6.73 | | 6.91 | 7.15 | 7.26 | 7.45 | | 7.64 | 8.11 | 8.82 | 0.13 | <0.001 |
| 18:2 *c*9*,t*11 | 0.05 | 0.05 | | 0.05 | 0.06 | 0.07 | 0.05 | | 0.08 | 0.04 | 0.05 | 0.01 | ns |
| 18:4 *c*6*,c*9*,c*12*,c*15 | 0.00 | 0.31 | | 0.38 | 0.44 | 0.53 | 0.62 | | 0.68 | 0.61 | 0.89 | 0.05 | <0.001 |
| 22:0 | 0.00 | 0.00 | | 0.02 | 0.02 | 0.01 | 0.01 | | 0.02 | 0.00 | 0.02 | 0.01 | ns |
| 20:4 *c*5*,c*8*,c*11*,c*14 | 1.15 | 1.16 | | 1.14 | 1.16 | 1.11 | 1.08 | | 1.11 | 1.07 | 1.11 | 0.04 | ns |
| 20:5 *c*5*,c*8*,c*11*,c*14*,c*17 | 0.37 | 0.39 | | 0.38 | 0.38 | 0.39 | 0.40 | | 0.39 | 0.40 | 0.42 | 0.02 | ns |
| unknown | 1.39 | 1.10 | | 0.99 | 0.90 | 1.12 | 1.29 | | 0.79 | 0.77 | 0.85 | 0.10 | ns |
| total SFA*^e^* | 8.39 | 6.69 | | 6.46 | 6.83 | 9.01 | 6.72 | | 7.18 | 6.13 | 6.08 | 0.43 | <0.01 |
| total MUFA*^f^* | 6.01 | 5.81 | | 5.47 | 5.54 | 5.75 | 5.32 | | 4.88 | 5.07 | 4.96 | 0.20 | <0.001 |
| total *trans* 18:1 | 0.51 | 0.48 | | 0.23 | 0.39 | 0.60 | 0.44 | | 0.34 | 0.25 | 0.39 | 0.04 | <0.05 |
| total PUFA*^g^* | 80.94 | 83.27 | | 83.98 | 83.65 | 81.20 | 83.85 | | 83.89 | 85.19 | 85.57 | 0.79 | <0.01 |
| total n-6 PUFA*^h^* | 73.79 | 75.76 | | 76.17 | 75.52 | 72.86 | 75.20 | | 74.97 | 75.94 | 75.29 | 0.80 | ns |
| total n-3 PUFA*^i^* | 7.04 | 7.42 | | 7.67 | 7.97 | 8.18 | 8.48 | | 8.72 | 9.11 | 10.13 | 0.15 | <0.001 |

*^a^*Means are based on 6 dairy cattle per treatment. *^b^*CON: control (0% of DM as lipid-encapsulated echium oil), *^c^*LEO: 1.5% of DM as lipid-encapsulated echium oil, 1.5% of DM as encapsulation, *^d^*HEO: 3% of DM as lipid-encapsulated echium oil. *^e^*Total SFA: sum of saturated fatty acids (4:0 to 26:0). *^f^*Total MUFA: sum of monounsaturated fatty acids (14:1 to 24:1). *^g^*Total PUFA: sum of polyunsaturated fatty acids (18:2 to 22:5). *^h^*Total n-6 PUFA: sum of n-6 PUFA: 18:2 *c*9*,c*12, 18:3 *c*6*,c*9*,c*12, 20:2 *c*11*,c*14, 20:3 *c*8*,c*11*,c*14, 20:4 *c*5*,c*8*,c*11*,c*14 and 22:4 *c*7*,c*10*,c*13*,c*16. *^i^*Total n-3 PUFA: sum of n-3 PUFA: 18:3 *c*9*,c*12*,c*15, 18:4 *c*6*,c*9*,c*12*,c*15, 20:5 *c*5*,c*8*,c*11*,c*14*,c*17, and 22:5 *c*7*,c*10*,c*13*,c*16*,c*19.
