# Supplementary material for: Lipid Encapsulation Provides Insufficient Total-Tract Digestibility to Achieve an Optimal Transfer Efficiency of Fatty Acids to Milk Fat
Source: PLoS One. 2016 Oct 14;11(10):e0164700. doi: 10.1371/journal.pone.0164700 (PMC5065208; doi:10.1371/journal.pone.0164700)
Supplement: S5 Table — (DOCX) [file pone.0164700.s006.docx]

**Supplemental Table 5.** Fatty acid composition*^a^* (g/100g FA) of the phospholipid plasma lipid fraction by day in response to CON*^b^*, LEO*^c^*, and HEO*^d^* diets.

| **fatty acid** | **Treatment** | | | | | | | | | | | **SE** | ***P* value** |
| --- | --- | --- | --- | --- | --- | --- | --- | --- | --- | --- | --- | --- | --- |
|  | **CON** | | **LEO** | | | | | **HEO** | | | |  |  |
|  | **D-**1 | **D**2  0**.**17  0**.**12  0**.**30  1**.**59  0**.**75**b**  1**.**33**a**  1**.**39**b**  0**.**70  0**.**00  23**.**1**b**  0**.**63**b**  0**.**09  0**.**40**ab**  0**.**81  2**.**64 | | **D**3  39**.**0  0**.**55  0**.**32  0**.**64  1**.**83**ab**  0**.**55  2**.**10  5**.**82  0**.**91  0**.**70  0**.**12  0**.**81  0**.**30  0**.**22  0**.**15 | **D**4  5**.**05**ab**  0**.**36**b**  0**.**02  0**.**19**a**  0**.**06  1**.**39**b**  0**.**09  0**.**18  0**.**08  0**.**00**a**  0**.**12**b**  0**.**18  0**.**41**ab**  0**.**23 | **D**7  0**.**17**a**  0**.**26  0**.**05**b**  0**.**10  1**.**29  67**.**6**ab**  17**.**82  5**.**98  8**.**60  5**.**87**ab**  1**.**87**a**  3**.**12**b**  0**.**35  4**.**51**a** | **D**9  0**.**18  0**.**06  0**.**24  1**.**47  0**.**63**c**  1**.**06**b**  1**.**23**c**  0**.**60  0**.**00  25**.**3**c**  0**.**54**b**  0**.**08  0**.**30**b**  0**.**70  2**.**24 | | **D**10  39**.**1  0**.**47  0**.**30  0**.**58  2**.**02**b**  0**.**55  1**.**99  5**.**33  0**.**85  0**.**70  0**.**11  0**.**88  0**.**33  0**.**23  0**.**19 | **D**11  4**.**30**b**  0**.**42**ab**  0**.**02  0**.**29**b**  0**.**09  1**.**56**c**  0**.**10  0**.**14  0**.**07  0**.**03**b**  0**.**23**c**  0**.**21  0**.**36**b**  0**.**16 | **D**14  0**.**25**b**  0**.**31  0**.**04**b**  0**.**17  1**.**33  69**.**4**b**  16**.**83  5**.**88  8**.**54  5**.**10**b**  2**.**28**b**  2**.**26**c**  0**.**37  3**.**77**b** |  |  |
| 10:0 | 0.00 | 0.32 | | 0.40 | 0.60 | 0.37 | 0.42 | | 0.37 | 0.37 | 0.15 | 0.04 | <0.01 |
| 12:0 | 0.00 | 0.00 | | 0.01 | 0.02 | 0.01 | 0.02 | | 0.03 | 0.01 | 0.02 | 0.00 | <0.01 |
| 13:0 | 0.00 | 0.01 | | 0.00 | 0.01 | 0.01 | 0.01 | | 0.01 | 0.01 | 0.00 | 0.00 | ns |
| *iso* 14:0 | 0.00 | 0.01 | | 0.01 | 0.01 | 0.03 | 0.04 | | 0.04 | 0.02 | 0.01 | 0.01 | <0.01 |
| 14:0 | 0.20 | 0.18 | | 0.13 | 0.14 | 0.15 | 0.15 | | 0.14 | 0.14 | 0.15 | 0.01 | <0.01 |
| *iso* 15:0 | 0.07 | 0.06 | | 0.07 | 0.07 | 0.06 | 0.06 | | 0.06 | 0.06 | 0.07 | 0.00 | 0.058 |
| *anteiso* 15:0 | 0.42 | 0.39 | | 0.39 | 0.34 | 0.35 | 0.35 | | 0.37 | 0.38 | 0.36 | 0.02 | ns |
| 15:0 | 0.39 | 0.34 | | 0.34 | 0.33 | 0.32 | 0.34 | | 0.36 | 0.34 | 0.32 | 0.01 | <0.01 |
| *iso* 16:0 | 0.21 | 0.20 | | 0.19 | 0.19 | 0.16 | 0.16 | | 0.16 | 0.17 | 0.17 | 0.01 | <0.01 |
| 16:0 | 13.43 | 13.18 | | 13.67 | 13.56 | 13.85 | 13.75 | | 13.78 | 13.98 | 13.96 | 0.26 | 0.075 |
| *iso* 17:0 | 0.34 | 0.34 | | 0.32 | 0.32 | 0.30 | 0.32 | | 0.30 | 0.32 | 0.30 | 0.02 | 0.067 |
| 16:1 *t*9 | 0.02 | 0.02 | | 0.02 | 0.02 | 0.02 | 0.03 | | 0.02 | 0.02 | 0.01 | 0.00 | ns |
| 16:1 *c*7 | 0.03 | 0.03 | | 0.02 | 0.03 | 0.03 | 0.03 | | 0.02 | 0.03 | 0.03 | 0.00 | ns |
| 16:1 *c*8 | 0.12 | 0.13 | | 0.13 | 0.10 | 0.10 | 0.08 | | 0.08 | 0.11 | 0.08 | 0.01 | <0.01 |
| *anteiso* 17:0 | 0.01 | 0.01 | | 0.02 | 0.02 | 0.01 | 0.02 | | 0.01 | 0.02 | 0.01 | 0.00 | ns |
| 16:1 *c*9 | 0.91 | 0.89 | | 0.86 | 0.81 | 0.76 | 0.72 | | 0.71 | 0.75 | 0.68 | 0.03 | <0.001 |
| 17:0 | 1.02 | 1.00 | | 0.97 | 0.97 | 0.93 | 0.92 | | 0.91 | 0.93 | 0.89 | 0.02 | <0.01 |
| *iso* 18:0 | 0.07 | 0.08 | | 0.08 | 0.06 | 0.05 | 0.05 | | 0.06 | 0.03 | 0.04 | 0.01 | <0.05 |
| 17:1 *c*8 | 0.13 | 0.12 | | 0.14 | 0.12 | 0.11 | 0.10 | | 0.09 | 0.09 | 0.09 | 0.01 | <0.01 |
| 17:1 *c*10 | 0.13 | 0.14 | | 0.13 | 0.10 | 0.11 | 0.10 | | 0.12 | 0.14 | 0.11 | 0.01 | ns |
| 18:0 | 25.40 | 25.34 | | 25.52 | 25.54 | 25.58 | 25.58 | | 25.67 | 25.60 | 25.70 | 0.18 | ns |
| 18:1 *t*5 | 0.01 | 0.01 | | 0.01 | 0.01 | 0.02 | 0.01 | | 0.01 | 0.01 | 0.00 | 0.01 | ns |
| 18:1 *t*6-8 | 0.05 | 0.09 | | 0.09 | 0.09 | 0.07 | 0.07 | | 0.06 | 0.08 | 0.07 | 0.01 | <0.01 |
| 18:1 *t*9 | 0.11 | 0.12 | | 0.10 | 0.10 | 0.10 | 0.09 | | 0.10 | 0.10 | 0.09 | 0.01 | ns |
| 18:1 *t*10 | 0.05 | 0.07 | | 0.06 | 0.07 | 0.07 | 0.07 | | 0.06 | 0.07 | 0.05 | 0.01 | ns |
| 18:1 *t*11 | 0.55 | 0.58 | | 0.57 | 0.57 | 0.61 | 0.67 | | 0.66 | 0.75 | 0.68 | 0.04 | <0.01 |
| 18:1 *t*12 | 0.19 | 0.30 | | 0.27 | 0.27 | 0.32 | 0.38 | | 0.35 | 0.33 | 0.29 | 0.32 | <0.05 |
| 18:1 *t*13/14/*c*6-8 | 0.53 | 0.61 | | 0.62 | 0.72 | 0.64 | 0.66 | | 0.65 | 0.63 | 0.52 | 0.02 | <0.05 |
| 18:1 *c*9 | 8.70 | 8.32 | | 8.48 | 8.15 | 7.26 | 6.79 | | 6.72 | 6.80 | 6.66 | 0.29 | <0.01 |
| 18:1 *c*11 | 0.62 | 0.60 | | 0.59 | 0.55 | 0.49 | 0.50 | | 0.47 | 0.51 | 0.45 | 0.03 | <0.01 |
| 18:1 *c*12 | 0.48 | 0.58 | | 0.50 | 0.48 | 0.45 | 0.48 | | 0.48 | 0.51 | 0.46 | 0.02 | ns |
| 18:1 *c*13 | 0.01 | 0.06 | | 0.03 | 0.03 | 0.02 | 0.03 | | 0.02 | 0.03 | 0.03 | 0.01 | 0.059 |
| 18:1 *c*14/*t*16 | 0.14 | 0.17 | | 0.17 | 0.16 | 0.14 | 0.15 | | 0.17 | 0.18 | 0.17 | 0.01 | <0.05 |
| 18:2 *t*10*,t*14 | 0.20 | 0.21 | | 0.18 | 0.15 | 0.14 | 0.13 | | 0.17 | 0.10 | 0.14 | 0.02 | <0.05 |
| cyclohexyl-11 11:0 | 0.25 | 0.27 | | 0.28 | 0.26 | 0.23 | 0.24 | | 0.23 | 0.27 | 0.20 | 0.03 | ns |
| 18:2 *c*12*,t*16 | 0.00 | 0.09 | | 0.10 | 0.07 | 0.05 | 0.06 | | 0.05 | 0.11 | 0.09 | 0.02 | <0.01 |
| 18:2 *c*9*,c*12 | 32.32 | 33.09 | | 32.26 | 32.65 | 33.96 | 34.36 | | 34.25 | 33.97 | 34.29 | 0.54 | <0.001 |
| 18:3 *c*6*,c*9*,c*12 | 0.25 | 0.41 | | 0.45 | 0.44 | 0.41 | 0.51 | | 0.55 | 0.60 | 0.61 | 0.03 | <0.001 |
| 18:3 *c*9*,c*12*,c*15 | 1.58 | 1.73 | | 1.82 | 1.91 | 1.86 | 2.00 | | 2.08 | 2.25 | 2.44 | 0.04 | <0.001 |
| 18:2 *c*9*,t*11 | 0.09 | 0.09 | | 0.07 | 0.08 | 0.07 | 0.07 | | 0.06 | 0.06 | 0.07 | 0.01 | <0.05 |
| 18:4 *c*6*,c*9*,c*12*,c*15 | 0.00 | 0.12 | | 0.13 | 0.14 | 0.13 | 0.16 | | 0.17 | 0.19 | 0.18 | 0.01 | <0.001 |
| 20:2 *c*11*,c*14 | 0.17 | 0.15  .10 | | 0.13 | 0.13 | 0.12 | 0.13 | | 0.13 | 0.13 | 0.14 | 0.01 | <0.01 |
| 22:0 | 0.02 | 0.02 | | 0.01 | 0.03 | 0.03 | 0.04 | | 0.04 | 0.03 | 0.04 | 0.01 | ns |
| 20:3 *c*8*,c*11*,c*14 | 4.78 | 4.35 | | 4.44 | 4.52 | 4.33 | 4.14 | | 4.13 | 3.96 | 3.97 | 0.11 | <0.001 |
| 20:4 *c*5*,c*8*,c*11*,c*14 | 2.24 | 2.11 | | 2.05 | 2.09 | 2.08 | 1.98 | | 1.95 | 1.93 | 1.96 | 0.08 | <0.001 |
| 23:0 | 0.05 | 0.04 | | 0.03 | 0.05 | 0.06 | 0.08 | | 0.07 | 0.04 | 0.05 | 0.01 | <0.05 |
| 20:5 *c*5*,c*8*,c*11*,c*14*,c*17 | 0.33 | 0.34 | | 0.32 | 0.32 | 0.37 | 0.35 | | 0.37 | 0.37 | 0.42 | 0.02 | <0.01 |
| 24:0 | 0.06 | 0.06 | | 0.03 | 0.04 | 0.07 | 0.08 | | 0.07 | 0.03 | 0.05 | 0.01 | ns |
| *cis*-5 24 :1 | 0.02 | 0.00 | | 0.01 | 0.01 | 0.03 | 0.03 | | 0.02 | 0.01 | 0.03 | 0.01 | ns |
| 22:4 *c*7*,c*10*,c*13*,c*16 | 0.53 | 0.48 | | 0.47 | 0.49 | 0.47 | 0.45 | | 0.46 | 0.44 | 0.43 | 0.03 | <0.01 |
| 22:4 *c*10*,c*13*,c*16*,c*19 | 0.13 | 0.10 | | 0.09 | 0.11 | 0.09 | 0.11 | | 0.10 | 0.11 | 0.11 | 0.01 | 0.051 |
| 22:5 *c*7*,c*10*,c*13*,c*16*,c*19 | 0.84 | 0.79 | | 0.74 | 0.76 | 0.76 | 0.75 | | 0.75 | 0.71 | 0.76 | 0.04 | <0.05 |
| 22:6 *c*4*,c*7*,c*10*,c*13*,c*16*,c*19 | 0.04 | 0.03 | | 0.03 | 0.03 | 0.03 | 0.03 | | 0.04 | 0.03 | 0.03 | 0.01 | ns |
| unknown | 1.60 | 1.32 | | 1.22 | 1.04 | 1.22 | 1.15 | | 1.26 | 1.82 | 1.38 | 0.09 | <0.01 |
| total SFA*^e^* | 40.58 | 40.46 | | 41.13 | 41.31 | 41.37 | 41.38 | | 41.44 | 41.52 | 41.34 | 0.30 | <0.01 |
| total MUFA*^f^* | 12.95 | 12.80 | | 13.00 | 12.45 | 11.34 | 11.01 | | 10.82 | 11.40 | 10.53 | 0.38 | <0.001 |
| total *trans* 18:1 | 1.20 | 1.18 | | 1.09 | 1.11 | 1.19 | 1.30 | | 1.25 | 1.34 | 1.18 | 0.08 | ns |
| total PUFA*^g^* | 43.45 | 44.09 | | 43.30 | 43.89 | 44.87 | 45.22 | | 45.25 | 44.99 | 45.64 | 0.54 | <0.001 |
| total n-6 PUFA*^h^* | 40.28 | 40.63 | | 39.81 | 40.31 | 41.37 | 41.58 | | 41.46 | 41.02 | 41.39 | 0.58 | <0.01 |
| total n-3 PUFA*^i^* | 2.91 | 3.08 | | 3.13 | 3.28 | 3.24 | 3.39 | | 3.51 | 3.65 | 3.95 | 0.05 | <0.001 |

*^a^*Means are based on 6 dairy cattle per treatment. *^b^*CON: control (0% of DM as lipid-encapsulated echium oil), *^c^*LEO: 1.5% of DM as lipid-encapsulated echium oil, 1.5% of DM as encapsulation, *^d^*HEO: 3% of DM as lipid-encapsulated echium oil. *^e^*Total SFA: sum of saturated fatty acids (4:0 to 26:0). *^f^*Total MUFA: sum of monounsaturated fatty acids (14:1 to 24:1). *^g^*Total PUFA: sum of polyunsaturated fatty acids (18:2 to 22:5). *^h^*Total n-6 PUFA: sum of n-6 PUFA: 18:2 *c*9*,c*12, 18:3 *c*6*,c*9*,c*12, 20:2 *c*11*,c*14, 20:3 *c*8*,c*11*,c*14, 20:4 *c*5*,c*8*,c*11*,c*14and 22:4 *c*7*,c*10*,c*13*,c*16. *^i^*Total n-3 PUFA: sum of n-3 PUFA: 18:3 *c*9*,c*12*,c*15, 18:4 *c*6*,c*9*,c*12*,c*15, 20:5 *c*5*,c*8*,c*11*,c*14*,c*17, and 22:5 *c*7*,c*10*,c*13*,c*16*,c*19.
