# Supplementary material for: Lipid Encapsulation Provides Insufficient Total-Tract Digestibility to Achieve an Optimal Transfer Efficiency of Fatty Acids to Milk Fat
Source: PLoS One. 2016 Oct 14;11(10):e0164700. doi: 10.1371/journal.pone.0164700 (PMC5065208; doi:10.1371/journal.pone.0164700)
Supplement: S7 Table — (DOCX) [file pone.0164700.s008.docx]

**Supplemental Table 7.** Fatty acid content of fecal matter (mg/g DM) from dairy cows*^a^* on CON*^b^*, LEO*^c^*, and HEO*^d^* diets.

|  | **Treatment** | | | **SE** | ***P*-value** |
| --- | --- | --- | --- | --- | --- |
|  | **CON** | **LEO** | **HEO** |  |  |
| total fatty acids | 21.09c | 65.63a | 55.21b | 2.59 | <0.001 |
| 14:0 | 0.27c | 0.75a | 0.61b | 0.04 | <0.001 |
| *iso*-15:0 | 0.13 | 0.11 | 0.12 | 0.01 | ns |
| *anteiso*-15:0 | 0.38a | 0.31b | 0.34b | 0.02 | 0.029 |
| 15:0 | 0.42 | 0.35 | 0.37 | 0.02 | ns |
| *iso*-16:0 | 0.15a | 0.09b | 0.11b | 0.01 | 0.002 |
| 16:0 | 3.45c | 28.60a | 22.20b | 1.04 | <0.001 |
| *iso*-17:0 | 0.22 | 0.17 | 0.19 | 0.02 | ns |
| 17:0 | 0.30 | 0.30 | 0.32 | 0.03 | ns |
| 18:0 | 5.67c | 26.61a | 22.21b | 1.63 | <0.001 |
| 18:1 *t*11 | 0.64b | 0.71b | 0.92a | 0.04 | <0.001 |
| 18:1 *c*9 | 1.14c | 1.56b | 2.06a | 0.07 | <0.001 |
| 18:1 *c*11 | 0.42a | 0.35b | 0.38b | 0.02 | 0.023 |
| 18:2 *t*9,*c*12 | 0.07c | 0.23b | 0.50a | 0.02 | <0.001 |
| 18:2 *c*9,*c*12 | 1.38b | 1.90a | 2.10a | 0.16 | 0.009 |
| 20:0 | 0.21b | 0.40a | 0.41a | 0.02 | <0.001 |
| 18:3 *c*6,*c*9,*c*12 | 0.00a | 0.14b | 0.30c | 0.01 | <0.001 |
| 18:3 *c*9,*c*12,*c*15 | 0.30c | 0.73b | 1.29a | 0.04 | <0.001 |
| 18:2 *c*9,*t*11 | 0.04b | 0.07a | 0.08b | 0.01 | 0.023 |
| 18:4 *c*6,*c*9,*c*12,*c*15 | 0.00c | 0.18b | 0.38c | 0.01 | <0.001 |
| 22:0 | 0.20b | 0.22ab | 0.22a | 0.01 | 0.038 |
| 20:5 *c*5,*c*8,*c*11,*c*14,*c*17 | 0.13c | 0.16b | 0.21a | 0.01 | <0.001 |
| 24:0 | 0.25 | 0.25 | 0.26 | 0.01 | ns |
| unknown | 0.08 | 0.06 | 0.07 | 0.01 | ns |
| total other*^d^* | 1.58 | 1.39 | 1.57 | 0.09 | ns |
| total SFA*^e^* | 14.53c | 57.67a | 44.73b | 2.48 | <0.001 |
| total MUFA**^f^** | 3.07b | 3.38b | 4.30a | 0.11 | <0.001 |
| total *trans* 18:1 | 1.04b | 1.07b | 1.35 | 0.08 | 0.003 |
| total PUFA*^g^* | 1.94c | 3.42b | 4.88c | 0.19 | <0.001 |
| total n-6 | 1.39b | 2.03a | 2.40a | 0.16 | 0.002 |
| total n-3 | 0.44c | 1.09b | 1.91a | 0.05 | <0.001 |

*^a^*CON: control (0% of DM as encapsulated echium oil),*^b^*LEO: 1.5% of DM as encapsulated echium oil, and 1.5% of DM as encapsulation matrix. *^c^*HEO: 3% of DM as encapsulated echium oil. *^d^*Ʃ Other: 12:0, *iso*-13:0, *iso*-14:0, 16:1 *t*9, 16:1 *c*7, 16:1 *c*8, , 16:1 *c*9, 16:1 *c*11, 17:1 *c*10, 18:1 *t*4, 18:1 *t*5, 18:1 *t*6-8, 18:1 *t*9, 18:1 *t*10, 18:1 *t*12, 18:1 *c*6-8/*t*13-14, 18:1 *c*12, 18:1 *c*13, 18:1 *c*14/*t*16, 18:1 *c*15, cyclohexyl-11:0, 20:0, 20:3 *c*11,*c*14,*c*17, 20:4 *c*5,*c*8,*c*11,*c*14, 23:0, 24:1 *c*15. *^e^*Total SFA: sum of saturated fatty acids (4:0 to 26:0). *^f^*Total MUFA: sum of monounsaturated fatty acids (14:1 to 24:1). *^g^*Total PUFA: sum of polyunsaturated fatty acids (18:2 to 22:5).
